# Proteome-wide identification of the endogenous ADP-ribosylome of mammalian cells and tissue

**DOI:** 10.1038/ncomms12917

**Published:** 2016-09-30

**Authors:** Rita Martello, Mario Leutert, Stephanie Jungmichel, Vera Bilan, Sara C. Larsen, Clifford Young, Michael O. Hottiger, Michael L. Nielsen

**Affiliations:** 1 Faculty of Health Sciences, Department of Proteomics, The Novo Nordisk Foundation Centre for Protein Research, University of Copenhagen, DK-2200 Copenhagen, Denmark; 2 Department of Molecular Mechanisms of Disease, University of Zurich, Zurich CH-8057, Switzerland; 3 Molecular Life Science Program of the Life Science Graduate School, University of Zurich, Zurich CH-8057, Switzerland

## Abstract

Although protein ADP-ribosylation is involved in diverse biological processes, it has remained a challenge to identify ADP-ribose acceptor sites. Here, we present an experimental workflow for sensitive and unbiased analysis of endogenous ADP-ribosylation sites, capable of detecting more than 900 modification sites in mammalian cells and mouse liver. In cells, we demonstrate that Lys residues, besides Glu, Asp and Arg residues, are the dominant *in vivo* targets of ADP-ribosylation during oxidative stress. In normal liver tissue, we find Arg residues to be the predominant modification site. The cellular distribution and biological processes that involve ADP-ribosylated proteins are different in cultured cells and liver tissue, in the latter of which the majority of sites were found to be in cytosolic and mitochondrial protein networks primarily associated with metabolism. Collectively, we describe a robust methodology for the assessment of the role of ADP-ribosylation and ADP-ribosyltransferases in physiological and pathological states.

## Abstract (web summary)

ADP-ribosylation is a reversible post-translational protein modification involved in many cellular processes. Here the authors describe a sensitive approach for the analysis of ADP-ribosylation sites under physiologic conditions and identify lysine residues as *in vivo* targets of ADP-ribosylation.

## Graphical abstract

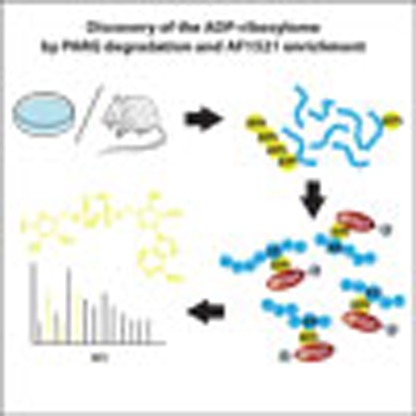

Protein ADP-ribosylation refers to the process where an ADP-ribose moiety is transferred from NAD^+^ to the amino acid side-chains of target proteins (as mono-ADP-ribose, MAR) or to an already protein bound ADP-ribose to form poly-ADP-ribose (PAR). These modifications are primarily catalysed by a class of enzymes known as ADP-ribosyltransferases (ARTs), with certain Sirtuin deacetylases also being able to catalyse ADP-ribosylation^1^. The ARTs can be divided further into two major subclasses: ARTCs (cholera toxin-like) and ARTDs (diphtheria toxin-like, formerly called poly(ADP-ribose) polymerases (PARPs)), depending on their conserved structural features^2^.

While MARylation has been reported to modulate GSK3β kinase activity and NF-κB signalling^3^, little is known about the biological functions of this type of modification. In contrast, PARylation has emerged as a crucial post-translational modification (PTM) in cancer development^4^. PARylation is a transient PTM^5^, whose rapid cellular degradation is predominantly carried out by PAR glycohydrolase (PARG)^6^. While PARylation is a key component of the DNA damage response (DDR) via its central role in the base excision repair pathway, many of the molecular details and processes affected by ARTs remain poorly understood. As a result, a detailed understanding of the molecular mechanisms and functions affected by ADP-ribosylation remains elusive.

In particular, the inventory of the amino acid residues modified by ADP-ribosylation remains incomplete. Current experimental evidence suggests that ADP-ribosylation primarily occurs on four different amino acids; Lys^7^, Arg^8^, Asp and Glu residues^9^. In addition, Cys residues were reported to be MARylated by certain ARTDs or bacterial toxins^10^. High-resolution mass spectrometry (MS) has become a valuable tool for comprehensive identification of PTMs^11^. However, current MS-based approaches for mapping ADP-ribosylation sites are biased towards modifications of only Glu and Asp^9^, or they lack sensitivity due to co-enrichment of other PTMs (that is, phosphorylated peptides)^12^.

Moreover, protein ADP-ribosylation is a low-abundant PTM that is rapidly degraded. To overcome this challenge cellular PARG knockdowns (siPARG) or knockouts have been developed^9^,^12^. Unfortunately, cellular absence of PARG leads to physiological alterations in cells, hepatocellular carcinoma in mice^13^, progressive neurodegeneration^14^ and excessive accumulation of PAR chains that are not rapidly degraded and promote cell death via parthanatos^15^. Consequently, strategies requiring knockdown of PARG constitute an improper setting for analysing physiological ADP-ribosylation and its associated mechanisms, thus rendering these methods inapplicable for *in vivo* analysis of tissues without genetic interventions^16^. Moreover, while ADP-ribosylation has been known for more than 50 years, the cellular stoichiometry of the modification has remained elusive, primarily due to the lack of methodologies that can elucidate such information^17^.

Recently a chemical genetic discovery method for ARTD targets was reported^18^, where the NAD^+^ analogue 8-Bu(3-yne)T-NAD^+^ was incubated with cell lysates from cells overexpressing mutated ARTDs sensitive to the analogue or cell lysates spiked with recombinant mutated ARTDs. However, as NAD^+^ is impermeable to the cell membrane, this method requires either the lysis of cells or the isolation of organelles (that is, nuclei) followed by the complementation of exogenous 8-Bu(3-yne)T-NAD^+^, which renders the identification of ARTD-specific substrates under different cellular conditions, and at physiological NAD^+^ levels unattainable. Moreover, the ADP-ribose acceptor sites identified using this methodology were limited to Glu and Asp modifications^9^.

To address these limitations, we have developed a protocol for the unbiased mapping of endogenous ADP-ribosylation sites in proteins. Our method led to the identification of more than 500 endogenous ADP-ribosylation sites in a single analysis and, as a result, provides an unprecedented in-depth analysis of protein ADP-ribosylation. Importantly, as the described workflow is applied under genetically unperturbed physiological conditions, we have used our methodology to analyse ADP-ribosylation sites in both cultured mammalian cells and mouse liver. Collectively, the workflow presented here represents a major advance in the detection of ADP-ribose acceptor sites and the identification of cellular processes regulated by ADP-ribosylation. Thus, facilitating a better understanding of the complex physiological and pathological processes that involve ADP-ribosylation, and the treatment of such conditions with PARP (that is, ADP-ribosylation) inhibitors.

## Results

### Identification of endogenous ADP-ribosylation sites

**Figure 1:**
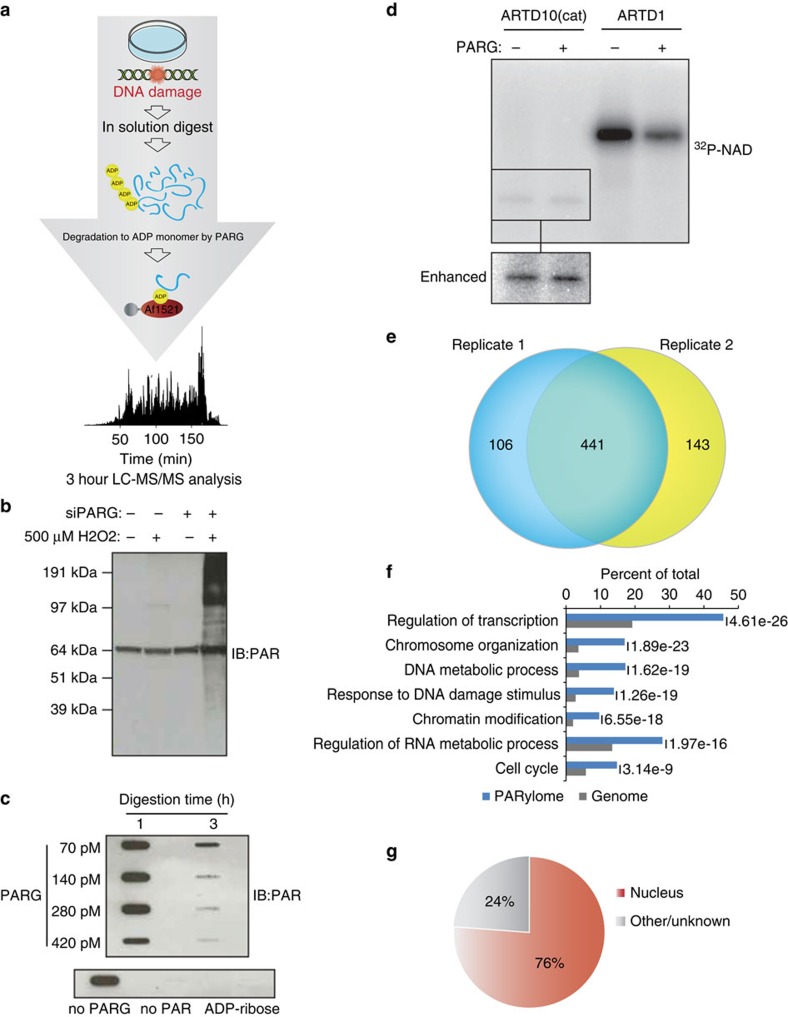
Proteome-wide identification of endogenous ADP-ribosylation sites in human cell culture. (**a**) Schematic representation of the peptide-based enrichment strategy. HeLa cells were treated with genotoxic stress and digested into peptides. Tryptic digested peptides were treated with PARG enzyme to convert multimeric ADP-ribosylation into monomeric counterparts, and subsequently ADP-ribosylated peptides were enriched using GST-Af1521 macrodomain. Enriched peptides were analysed by high-resolution LC-MS/MS on an Orbitrap Q-Exactive HF instrument and the data was further processed by bioinformatic software tools. (**b**) Comparison of HeLa cells exposed with 500 μM H_2_O_2_. During cellular knock-down of PARG enzyme (siPARG) an abundant PAR signal is observed, while under physiological conditions the PAR signal is significantly weaker (compare second lane with fourth lane on gel). Previous methods for characterizing ADP-ribosylation solely worked under siPARG conditions while the methodology described here is applicable to physiological conditions. (**c**) Optimization of the incubation time and amount of PARG enzyme required for converting multimeric ADP-ribosylated peptides into monomeric counterparts. (**d**) Validation experiment that confirms PARG treatment does not remove MAR from investigated peptides. (**e**) Venn diagram of identified ADP-ribosylation sites identified in two biological replicate analyses. A strong overlap in identified sites signifies high reproducibility in the developed method. (**f**) GO functional annotation of significantly regulated proteins in the combined data set reveal strong enrichment of proteins involved in DNA repair processes compared with annotated GO genes across the entire human genome (indicated *P*-values<0.005). (**g**) GO term annotation enrichment for cellular distribution of proteins harbouring ADP-ribosylation sites.

We have developed a technology for sensitive analysis of endogenous ADP-ribosylation sites in both cells and tissues that overcomes several of the above-mentioned limitations of current approaches. Briefly, proteins are isolated from cells, digested into peptides first using LysC and then trypsin. We then treat the cellular peptide digest with PARG, thereby converting all PARylated amino acids to their MARylated counterparts^19^. While this prevents discriminating whether the modification was originally PARylation or MARylation, the conversion is crucial for feasible MS analysis. Furthermore, this has the advantage that MARylated peptides can be unbiasedly enriched with an ADP-ribose-specific domain^20^. In contrast to previously described methodologies^21^, enrichment at the peptide level with Af1521 in combination with prior PARG treatment has not been performed before. Subsequently, the enriched ADP-ribosylated peptides and their acceptor sites are identified using a high-resolution Orbitrap mass spectrometer (Q Exactive HF). As no pre-fractionation steps are employed, the described workflow analyses ADP-ribosylated peptides from a single sample requiring only a few hours of sensitive LC-MS operation^22^. All peptides are fragmented using higher-energy collisional dissociation (HCD) ensuring high p.p.m. accuracy on both the precursor and fragment ions^23^. Furthermore, ADP-ribosylated peptide identification is aided by diagnostic ions originating from the fragmentation of the ADP-ribose group linked to the peptide^24^,^25^ (Supplementary Fig. 1a). Superior advancements over current methodologies are as follows: First, sample preparation without ARTD, NAD^+^ or PARG level perturbation^9^,^18^ allows analysis of both cells and tissues under physiological conditions. This will allow us to broaden our understanding of the mammalian ADP-ribosylation complexity, and facilitating comparisons across any cellular condition, cell type and, even, species. To substantiate this, we have applied our established method to HeLa cells exposed to hydrogen peroxide (H_2_O_2_)-induced oxidative stress (Fig. 1a), and to normal mouse liver samples. Second, using the Af1521 macro domain to enrich ADP-ribosylated peptides, which has favourable binding preferences and relatively high ADP-ribose affinity with a Kd of ∼0.13 μM (ref. 26), allowed unbiased modified amino acid analysis. Third, we have ensured prevention of lysis-induced ADP-ribosylation artefacts, as previously reported^20^, thus facilitating assessment of the ADP-ribosylome during actual physiological conditions.

### Identification of the H_2_O_2_-induced ADP-ribosylome

To benchmark our methodology, we treated HeLa cells with 500 μM H_2_O_2_, which induces PAR formation through oxidative stress signalling. Under physiological conditions (non-siPARG conditions), H_2_O_2_ treatment induces PAR levels only faintly detectable by immunoblot (Fig. 1b), while cellular knock-down of PARG via small interfering RNA (siPARG) causes strong PAR formation (Fig. 1b). This demonstrates the requirement for improved sensitivity in studying ADP-ribosylation under unperturbed physiological conditions.

To ensure complete catalysis of PARylated into MARylated peptides we next assessed the amount of PARG required to catalyse a fixed amount of PAR. Previously, PAR levels in HeLa cells have been reported to range up to 0.1 amol per cell during H_2_O_2_ treatment^27^. Thus, we incubated 10 μM of purified PAR with increasing concentrations of PARG and assessed the temporal efficiency of the catalysis by immune-slot blot (Fig. 1c). Since the most efficient catalysis was observed when PAR was treated with PARG for 3 h, we concluded that this enzyme ratio allowed enzymatic catalysis of PARylated peptides into MARylated peptides during our enrichment procedure.

As some macrodomains can exhibit hydrolase activity on MAR moieties^28^,^29^, we performed the Af1521 enrichment procedure at 4 °C and incubated samples for only two hours. Moreover, all subsequent sample handling steps were performed at 4 °C, which collectively prevents hydrolase activity of the Af1521 macro domain^20^. To confirm that PARG exerted no enzymatic hydrolase activity on MAR (ref. 19) in our workflow, we assessed whether PARG removes ADP-ribosylation from automodified ARTD1 (also PARP1) or ARTD10 (formerly PARP10; Fig. 1d), which are known PARylated and MARylated substrates^30^, respectively.

Using autoradiography assays, hydrolysis of the attached radioactive [^32^P]-PAR of the protein substrates was monitored (Fig. 1d), revealing that PARG was indeed able to convert PAR chains on ARTD1 into MAR, while no reaction was observed regarding the auto-MARylation of ARTD10 (Fig. 1d). These results confirmed that PARG hydrolase activity would not affect enrichment of MAR residues.

To evaluate the reproducibility of the methodology, we performed replicate enrichment experiments in HeLa cells. Following MS analysis, there was a 75% overlap of the high-confident ADP-ribose acceptor sites identified (localization scores >0.60) between replicates (Fig. 1e). Thus, this indicates high reproducibility in the established approach, which compares well with the reproducibility obtained in other proteomic experiments (Supplementary Note 1)^31^. From three replicates, we have identified 739 ADP-ribosylation sites (Localization score >0.60) on 480 proteins after H_2_O_2_ treatment (Supplementary Data 1), with the majority of identified proteins containing a single modification site (Supplementary Fig. 1b). To corroborate our identification analysis, we performed a separate MS analysis employing the complementary fragmentation technique electron transfer dissociation (ETD)^32^, which confirmed the localization of several identified sites (Supplementary Fig. 1c,d; Supplementary Data 2).

Functional analysis using gene ontology (GO) confirmed that the identified proteins participate in biological processes known to involve ADP-ribosylation activity, including transcription, chromosome organization and response to DNA damage stimulus^33^,^34^ (Fig. 1f). Reassuringly, we find that 76% of the ADP-ribosylated proteins localize to the nucleus (Fig. 1g), in line with the cellular localization of ARTD1 and ARTD2 (also PARP2). Moreover, the cellular abundance profile of identified ADP-ribosylation protein targets supports the notion that our methodology is not biased towards abundant proteins (Supplementary Fig. 1e). Collectively, these results confirm the feasibility and reproducibility of this novel proteomics approach for identifying the endogenous ADP-ribosylation sites in cultured HeLa cells.

### Identification of Lys residues as endogenous acceptor sites

**Figure 2:**
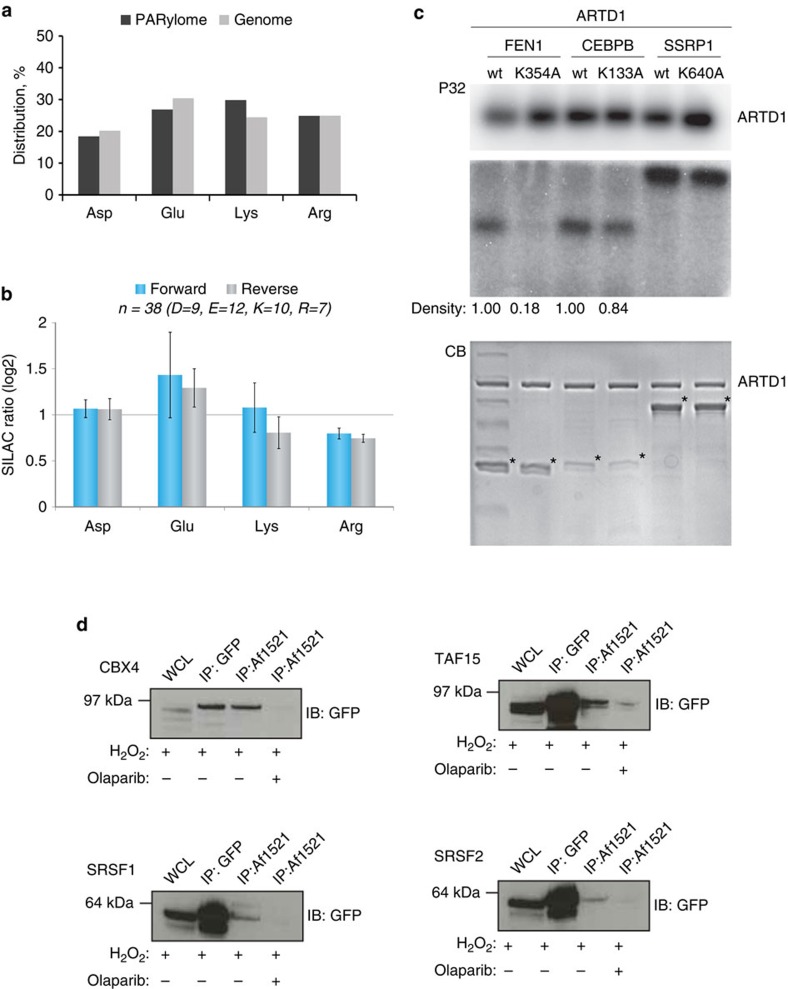
Lysine residues are *in vivo* targets of ADP-ribosylation in human cells. (**a**) Distribution of ADP-ribosylation acceptor sites compared with their distribution in the genome. (**b**) Assessment of peptide glycation by free ADP-ribose. Distribution of log2 transformed SILAC ratios, and ADP-ribosylation acceptor sites, from forward and reverse SILAC experiments as outlined in Supplementary Fig. 2a. No increased SILAC ratios were observed for the different acceptor sites when cells were treated with PAR, supporting the notion, that the observed modifications are not derived from glycation. (**c**) *In vitro* PARylation of identified protein targets. Purified full-length human ARTD1 was incubated with recombinantly expressed proteins in the presence of ^32^P-NAD^+^ and double-stranded DNA oligomer. Samples were resolved by SDS–PAGE, stained with Coomassie (CB; lower panel) and ^32^P-incorporation was detected by autoradiography (P32; upper panel). (**d**) HeLa cells stably expressing SRSF1, SRSF2 CBX4 or TAF15 as GFP-fusion proteins were treated with H_2_O_2_ and PARP (that is, ADP-ribosylation) inhibitor olaparib as control experiment. Lysates were subjected to Af1521 WT pull-down or GFP-immunoprecipitation and subsequently analysed by immunoblotting with GFP antibody. Error bars are 95% confidence intervals with *n*=4.

As the Af1521 macro domain binds to the ADP-ribose moiety^26^ our methodology allows for unbiased detection of any ADP-ribose acceptor site. In support of this, no discernable difference was observed when the amino acid distribution of ADP-ribosylation sites was compared with the amino acid distribution of the same residues across the proteome (Fig. 2a). Among the identified ADP-ribosylation sites, we observed a significant portion of modifications residing on Lys residues, including the previously confirmed ARTD1 modification site K498 (ref. 7). ADP-ribosylation of Lys residues has been suggested as an artefact related to the release of ADP-ribose moieties during PARG cleavage of PAR chains^17^. This suggestion was made based on observations that ADP-ribose was found to non-enzymatically attach to Lys, Arg and Cys residues in a glycation process^35^. However, the incorporation rate (stoichiometry) achieved in this study, which utilized large amounts of histone proteins for *in vitro* reactions, were estimated to be below 2%, suggesting that this is an inefficient reaction^35^. To investigate whether PARG treatment of PAR chains causes glycation in our experimental setup, we performed a quantitative experiment using Stable Isotope Labelling by Amino acids in Cell culture (SILAC)^36^. Herein ‘Heavy' SILAC cell lysates were treated with free PAR chains before PARG degradation, while ‘Light' SILAC cell lysates were left untreated (Supplementary Fig. 2a). Since no PAR-inducing stress was exerted on these cells, identification of ADP-ribosylation sites exhibiting increased SILAC ratios would be indicative of chemical reactions caused by free ADP-ribose released by PARG. Here we identified 39 ADP-ribosylation sites equally distributed across Lys, Arg, Glu and Asp residues, and no increased SILAC ratios were observed for identified modification sites. These findings strongly suggest that PARG treatment does not lead to random glycation of Lys or Arg residues (Fig. 2b and Supplementary Data 3). To substantiate our findings, we performed a ‘reverse' SILAC experiment where only light SILAC cells were treated with free PAR chains, which resulted in a similar outcome (Fig. 2b). These results confirm that ADP-ribose moieties released on PARG treatment are unlikely to cause relevant *in vitro* artefacts and, combined with the overall reproducibility of ADP-ribosylation site identification (Fig. 1e), suggest that the identified ADP-ribosylation sites were not derived from non-enzymatic glycation.

In addition, an *in vitro* experiment utilizing a synthesized histone H2B-like peptide (NH_2_-PQPAKSAPAPKKG-OH) incubated with free ADP-ribose was performed analogous to previously reported experiments^35^. Briefly, the non-modified H2B peptide was incubated with 1 mM ADP-ribose at 37 °C at pH 9 or 7.5 for two time points (1 h or overnight incubation). Glycation levels were then determined using time-of-flight (TOF) MS (Supplementary Fig. 2b). On incubation with free ADP-ribose, only small levels of glycation were observed, dependent on pH and incubation time (Supplementary Fig. 2b). Tandem mass spectrometry (MS/MS) confirmed that glycation took place at Lys residues (Supplementary Fig. 2c), corroborating earlier observations that free ADP-ribose is able to modify Lys residues by non-enzymatic glycation. However, our data reveal that glycation occurs primarily at high pH and requires non-physiological concentrations of free ADP-ribose. In contrast, PARG-released ADP-ribose was not able to induce similar artefacts at detectable levels. Moreover, the non-enzymatic glycation of ADP-ribose occurred primarly on several Lys residues within the short H2B-peptide, which is in stark contrast to the different ADP-ribosylation sites observed in cell culture (Supplementary Fig. 1b). Collectively, these results strongly suggest that the ADP-ribosylation sites observed in our cell culture analysis were not caused by glycation.

To further validate Lys residue ADP-ribosylation *in vivo*, we biochemically confirmed the Lys modification sites identified on FEN1, CEBPB and SSRP1 in cells using an *in vitro* PARylation assay. To this end, recombinant proteins for these target substrates were purified as wild type (WT) and potentially modification-deficient mutant variants, with the latter harbouring K-to-A mutations at Lys residues that we found to be modified (K354A for FEN1, K133A for CEBPB and K640A for SSRP1) (Supplementary Data 1). All proteins were incubated with purified ARTD1 in the presence of [^32^P]-NAD^+^ and a DNA fragment to measure the incorporation of NAD^+^ radioactivity by autoradiography^37^. Activation of ARTD1 was confirmed by a strong automodification (Fig. 2c, upper panel). Using ARTD1 we detected strong ADP-ribosylation signals for all three WT protein candidates, confirming that these are ADP-ribosylation substrates. When Fen1, CEBPB or SSRP1 were trans-modified with either ARTD1, ARTD1+PJ34, ARTD1 Y907A/C908R (catalytically dead) or ARTD1 E988K (1.25% of wild-type activity; only monomers are added), modification of the substrates was not observed (Supplementary Fig. 3), confirming that glycation is not a problem in our *in vitro* assays.

However, in our data set we observed several other ADP-ribosylation sites in this protein, although these had lower localization scores. These findings suggest that the K640 modification may only contribute a low percentage of the total ADP-ribosylation levels for SSRP1. Conversely, for the K133A mutant of CEBPB9, we observed a 16% decrease in the ADP-ribosylation signal. Analogously, when K354 was mutated to Ala in FEN1, a reduction in total PAR signal was also observed, thus confirming that this residue constitutes an ADP-ribosylation site. Altogether, these findings demonstrate that Lys residues are indeed specific targets of ADP-ribosylation. In addition, we confirmed SRSF1, SRSF2, TAF15 and CBX4 as *in vivo* ADP-ribosylated protein substrates using western blot (WB) analysis (Fig. 2d). These targets were selected for three reasons: TAF15 is a known ADP-ribosylated substrate during oxidative stress^9^,^20^ and serves to demonstrate the ability of the methodology to confirm known targets. For SRSF1, the identified modification sites were observed with localization scores of 0.5, and therefore do not constitute *bona fide* high-confident sites (Supplementary Data 1). Importantly, we demonstrate that the data obtained could still be used to infer that SRSF1 is an ADP-ribosylation target substrate as WB confirmed the MS results. Finally, both SRSF2 and CBX4 are novel ADP-ribosylation targets harbouring high-confident modifications sites.

### ADP-ribosylation and PAR formation dynamics correlate

**Figure 3:**
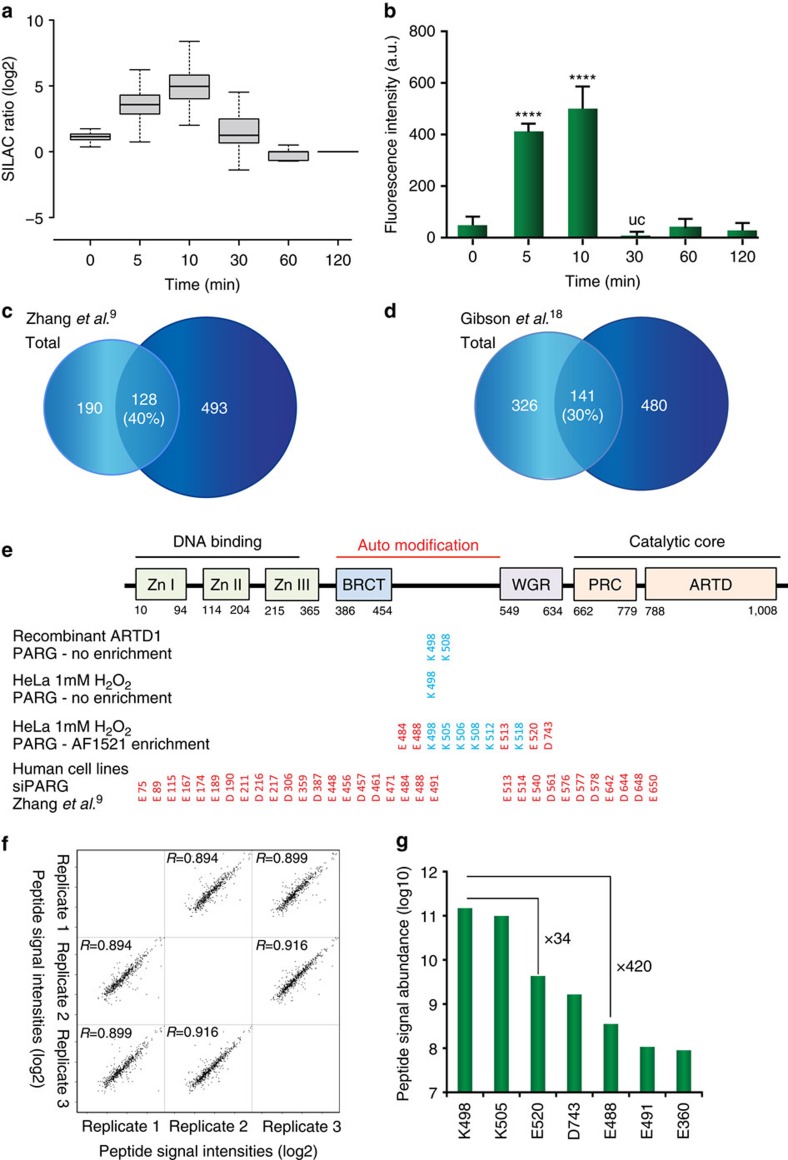
SILAC ratios of ADP-ribosylation sites. (**a**) Boxplot analysis of logarithmized H/L SILAC ratios from six SILAC experiments representing HeLa cells treated with H_2_O_2_ in a temporal manner (see Supplementary Fig. 2a). Strongest regulation of ADP-ribosylation sites is observed when cells are treated for 5–10 min of genotoxic stress. (**b**) Densiometric evaluation of IF analysis of HeLa cells treated with H_2_O_2_ for different time points. The strongest abundance in PAR signal is observed after 5–10 min treatment of H_2_O_2_, in good correlation with observed increase in SILAC ratios on MS analysis (Fig. 2a). Experiments were performed in triplicates. (**c**) Venn diagram depicts overlap between identified ADP-ribosylated proteins compared with previously reported Asp and Glu ADP-ribosylated proteins. (**d**) Venn diagram depicts overlap between identified ADP-ribosylated proteins compared with previously reported Asp and Glu ADP-ribosylated proteins using an *in vitro* strategy. (**e**) Comparison of identified ARTD1 ADP-ribosylation sites across different experiments as indicated. (**f**) Multi-scatter plot of measured peptide signal intensities from triplicate ADP-ribosylation experiments. A strong Pearson correlation signifies high reproducibility in the measured abundance of ADP-ribosylated peptide species. (**g**) Abundance measurement for seven ADP-ribosylation sites, demonstrating that lysine residue K498 is abundantly modified in ARTD1. Error bars are 95% confidence intervals with *n*=3.

Since the established enrichment approach requires PARG enzymatic conversion of ADP-ribosylated acceptor sites into their MARylated counterparts, we examined the dynamics of the H_2_O_2_-induced ADP-ribosylation sites identified using SILAC. For this, Light SILAC cells were stimulated for only a few seconds with 500 μM H_2_O_2_ (∼0 min), while heavy SILAC cells were treated with the same concentration of H_2_O_2_ for various durations (0, 5, 10, 30, 60 and 120 min) (Supplementary Fig. 4a). Using this approach, we investigated the effect oxidative stress (H_2_O_2_ treatment) has on the abundance of ADP-ribosylation sites determined by quantitative MS: if the identified ADP-ribosylation site is induced on oxidative stress, then the relative SILAC peptide intensity ratio between light and heavy peaks will be higher in the heavy isotope encoded sample, thereby exhibiting an increased SILAC ratio. Following MS analysis of H_2_O_2_-treated SILAC samples, we extracted and compared the temporal SILAC ratios of identified ADP-ribosylation sites, and observed the highest SILAC ratios at early time points (5 and 10 min H_2_O_2_ treatment) (Fig. 3a). To examine whether changes in SILAC ratios correlate with the dynamics of cellular PAR formation, we compared the SILAC readout signal (Fig. 3a) to the PAR signals analysed by immunofluorescence in the same cells. Although the employed antibody primarily recognizes only longer PAR chains, the employed approach constitutes a widely used methodology to evaluate relative differences (that is, dynamic changes) in cellular PAR formation^38^,^39^,^40^ (Fig. 3b; Supplementary Fig. 4b). From triplicate IF experiments a good temporal and kinetic correlation between the measured SILAC ratios (Fig. 3a) and PAR IF signals was observed (Fig. 3b), with both analyses exhibiting the highest increase at 5–10 min of H_2_O_2_ treatment. Moreover, the dynamic changes observed are similar to results obtained from immuno-slot-blot analysis of PAR formation (Supplementary Fig. 4c), and to those reported in mouse embryonic fibroblasts treated with 100 μM H_2_O_2_. Also in these experiments a peak of PAR formation was observed after 5 min, with subsequent turnover after 15–20 min (ref. 39). Collectively, these data show that the upregulation of ADP-ribosylation sites determined by SILAC ratios correlates with the increase in PAR formation using IF analysis.

### Determination of endogenous ADP-ribosylation stoichiometry

Today, large-scale proteomics experiments have been very successful in determining the relative abundance of PTMs between different cellular states^11^. However, an inherent challenge in PTM analyses is the estimation of stoichiometry, referred to as the fraction of a given protein modified with a particular PTM at a given amino acid. To obtain stoichiometry information, we used the information gathered in our H_2_O_2_-treated SILAC experiments (Fig. 3a and Supplementary Data 1), and combined it with data characterizing general protein regulation during H_2_O_2_ treatment^41^. Briefly, ADP-ribosylated peptides have opposite ratios of their unmodified counterparts, which can be used to calculate the absolute stoichiometry of modified sites from any two SILAC states. This calculation is made under the assumption that the sum of modified and unmodified peptides remains constant between SILAC states^41^. From a single experiment, we obtained stoichiometry values for 55 ADP-ribosylation sites, revealing that half of the ADP-ribosylation sites have less than 11% stoichiometry on H_2_O_2_ treatment (that is, the fraction of a given modification site occupied by ADP-ribosylation; Supplementary Fig. 4d). In line with the overall transient nature of the modification, these findings suggest a tight enzymatic regulation of ADP-ribosylation stoichiometry. Moreover, several of the arginine residue ADP-ribosylation sites were measured with high stoichiometry (Supplementary Fig. 4e, Supplementary Data 4). Whether these represent protein targets modified by ARTs other than ARTD1 and 2, or represent MARylation rather than PARylation remains to be determined.

### Comparing this ADP-ribosylome to Asp/Glu ADP-ribosylomes

With the wide range of ADP-ribosylated proteins identified, we sought to compare our list of modified proteins with the previously reported proteins ADP-ribosylated at Asp/Glu (ref. 9). Although these analyses were conducted in different cell lines and under different physiological conditions, we found that 36 per cent of the reported Asp/Glu ADP-ribosylated proteins were also modified in our data set (Fig. 3c). Similarly, 38% of the targets identified in our previous report using the Af1521 domain for identification of ADP-ribosylated proteins^20^ were also identified with the current methodology (Supplementary Fig. 4f). This overlap increased to 52 percent when only H_2_O_2_-induced ADP-ribosylation substrates were compared, supporting the notion that differences in the substrates identified do not stem from the methodologies but from differences in cellular conditions (Supplementary Fig. 4f). Similarly, when comparing our data set with an *in* *vitro* analysis where 8-Bu(3-yne)T-NAD^+^ was incubated with cell lysates and mutated analogue-sensitive ARTDs^42^ an overlap of only 30% was observed (Fig. 3d). These findings suggest that *in vivo* and *in vitro* strategies target different ARTD substrates.

To further investigate the comparability of the different enrichment strategies, we performed SILAC experiments in which the PARP inhibitor olaparib was introduced before oxidative stress and compared the outcome with the analogous ADP-ribosylome experiment^9^. In two SILAC experiments, both light and heavy SILAC cells were treated with H_2_O_2_, while only light SILAC cells were treated with olaparib (1 μM or 10 μM)^43^. A strong Pearson correlation in SILAC ratios between the experiments (*R*=0.69) signifies that the ADP-ribosylation sites identified were similarly affected by the employed olaparib concentrations (Supplementary Fig. 4g). Using the STRING database of physical and functional interactions^44^, we found that the proteins harbouring ADP-ribosylation sites regulated by olaparib were strongly connected with ARTD1 and ARTD2 (Supplementary Fig. 5a). Moreover, the regulated proteins were also strongly associated with biological processes known to involve ARTDs (Supplementary Fig. 5b) and therefore most probably constitute ADP-ribosylated candidates.

Next, we compared the protein distribution of identified modification sites between this data set and the above-mentioned methodology. In the Asp/Glu ADP-ribosylome^9^, a total of 1,048 ADP-ribosylation sites residing on 320 proteins were identified, which corresponds to 3.3 modifications per identified substrate. In contrast, our combined data set includes 958 ADP-ribosylation sites on 565 proteins, or 1.7 modifications per identified substrate. Considering that only half of the identified sites in our data set reside on Glu or Asp residues (Fig. 2a), the boronic acid approach, which employs non-physiological siPARG conditions, identifies more ADP-ribosylation sites per identified substrate. Notably, this increase is analogous to the observed increase in overall PAR signal on siPARG treatment (Fig. 1b).

We then compared the ADP-ribosylation sites only identified on ARTD1. In the Asp/Glu ADP-ribosylome analysis by Zhang *et al*.^9^, a total of 37 ADP-ribosylation sites were reported for ARTD1 (Fig. 3e), of which 23 resided on Glu residues, which corresponds to 31% of the total number of Glu within human ARTD1. In contrast, the Af1521 analysis only identified ADP-ribosylation in a total of 11 amino acid acceptor sites across all experiments (Fig. 3e). To investigate whether the differences in the number of identified ADP-ribosylation sites might be abundance-driven, we performed an analysis of HeLa lysates without Af1521 enrichment. In addition, we performed a similar analysis with recombinant ARTD1, where automodified ARTD1 was treated with PARG but not enriched by Af1521. From these experiments, we solely found K498 to be modified in the non-enriched HeLa sample, whereas both K498 and K505 were identified on recombinant ARTD1 (Fig. 3e). These data suggest that certain Lys residues within the auto-modification domain of ARTD1 are most abundantly present in the analysed samples, which is similar to previous mutational observations for ARTD1 (ref. 7). We observed a strong reduction in the *in vitro* PAR signal when the three lysine residues within the automodification domain of ARTD1 (K498R, K521R and K524R)^7^ were mutated. Thus, indicating that these sites are indeed relevant for automodification of ARTD1 and supporting our observations that K498 is a major PAR acceptor site of ARTD1.

To quantify ARTD1 modification sites in more detail, we next compared the peptide signal abundance between replicate Af1521 analyses (Fig. 1e). To this end, the intensity values for all identified peptide sequences were compared across replicate samples. A strong Pearson correlation between replicates (*R*>0.89) demonstrates that the measured peptide signal intensities can be used as a reliable measure for modification site abundance (Fig. 3f). To further investigate quantification of ADP-ribosylation sites, we performed abundance assessment for seven modification sites on ARTD1 that were reliably identified in two out of the three replicate experiments (Fig. 3g). The analysis revealed that ADP-ribosylation located on lysine K498 yielded the strongest signal abundance. Although peptides from the same protein might exhibit different ‘flyability'^45^, an observed 34-fold and 420-fold difference in signal intensity compared with nearby modification sites residing on glutamic acids E520 and E488, respectively, suggests that K498 is an abundant auto-modification site on ARTD1. Besides, ADP-ribosylation on K498, both E488 and E491 reside on comparable tryptic peptide sequences within ARTD1, so that the observed differences in abundance cannot be attributed to different peptide ionization propensities. We observed that quantified ADP-ribosylation sites preferentially reside within the auto-modification domain of ARTD1 under physiological conditions (Fig. 3g,e).

### Analysing endogenous ADP-ribosylation sites in mouse liver

**Figure 4:**
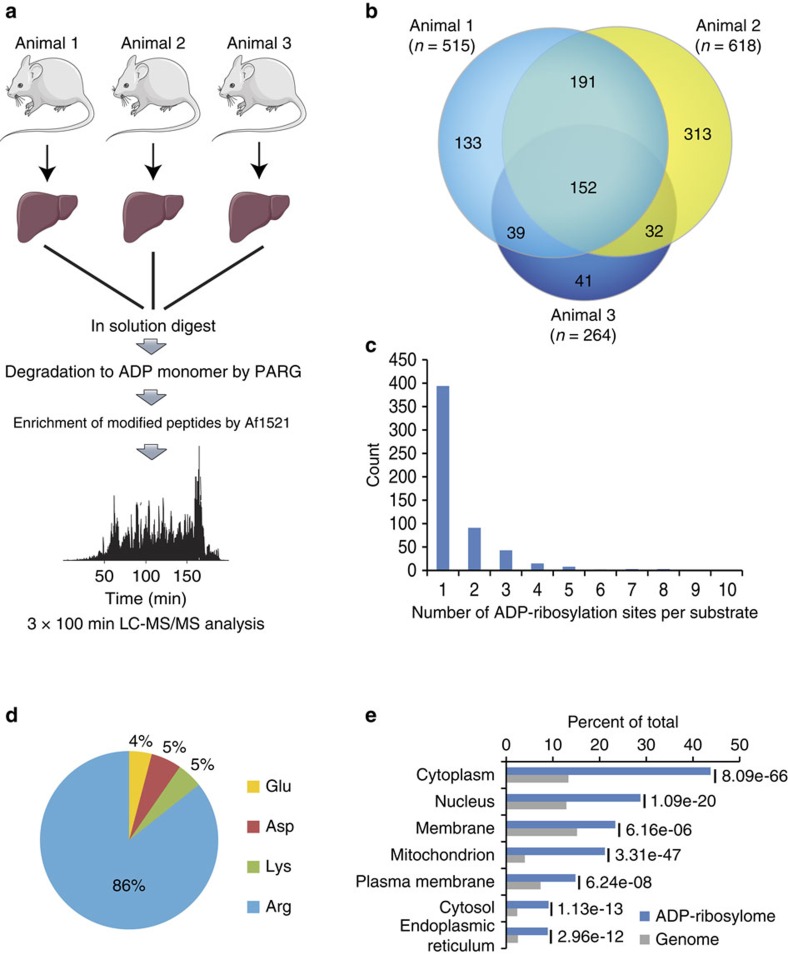
Proteome-wide identification of endogenous ADP-ribosylation sites in mammalian tissue. (**a**) Experimental setup for mammalian liver tissue analysis. In total, liver samples derived from three mice were investigated and prepared as indicated. Mouse images were adapted from the Servier Image Bank under the Creative Commons licences CC-BY. (**b**) Venn diagram of identified ADP-ribosylation sites from three liver samples. A strong overlap between analysed sampled signifies good reproducibility in the identified ADP-ribosylation sites across investigated tissue samples. (**c**) Distribution of ADP-ribosylation sites across proteins. (**d**) Distribution of ADP-ribosylated amino acids. The majority (86%) of identified sites reside on arginine residues indicative of differential ART activity compared with cell culture analysis (Fig. 1g). (**e**) GO term annotation enrichment for cellular distribution of proteins identified in liver samples harbouring ADP-ribosylation sites.

Tissues can contain many different cell types that display a wide range of protein concentrations, which poses challenges to proteomic identification and the analysis of PTMs. Moreover, many tissues contain a broad range of different mono-ARTs, including members of the ARTD family, SIRTs and ARTCs (ref. 46). To further explore the general applicability of the new method, we characterized the endogenous ADP-ribosylome of mouse liver, a tissue that has already been described to regulate cellular processes in an ADP-ribosylation-dependent manner^47^. Three C57BL/6 mice were killed before their livers were collected and frozen in liquid nitrogen. The tissue was then ground up and processed as described for the HeLa cells (Fig. 4a). In triplicate analyses, we identified 901 modified peptides with unique ADP-ribosylation acceptor sites, of which 414 were identified in at least two different liver samples (Fig. 4b, Supplementary Data 5). The distribution of ADP-ribosylation sites was similar to the cell culture analysis, with 70% of identified proteins harbouring only one modification site (Fig. 4c). Strikingly, the majority (86%) of identified ADP-ribosylation acceptor sites in the mouse liver were Arg, while Lys, Asp and Glu were detected only at very low levels compared with HeLa cells (Fig. 4d). Notably, several Lys residues modified with ADP-ribosylation were also found on cytoplasmic proteins in this analysis (Supplementary Data 5), providing evidence that lysine residues being *in vivo* targets of ADP-ribosylation under physiological conditions.

GO analysis of the modified proteins revealed a high enrichment in mitochondrial, cytoplasmic, nuclear and membrane proteins (Fig. 4e). Among the identified ADP-ribosylated proteins, we found several previously reported ADP-ribosylated proteins. These included ARTD12 (formerly PARP12), which has been described to have MAR and auto-ADP-ribosylation activity; ARTC2, which is a GPI-anchored arginine-specific MARylating enzyme that can be shed and circulated in the blood; and glutamate dehydrogenase 1, which is known to be regulated through ADP-ribosylation by SIRT4 (refs 48, 49). In agreement with experiments done in HeLa cells, we also found that histone H2B and several RNA helicases were modified. These results indicate that the established enrichment protocol can readily be employed to investigate the non-induced ADP-ribosylomes of tissues, which include both intra- and extra-cellular ADP-ribosylated proteins.

## Discussion

Here, we describe the establishment of a robust and highly reproducible technology for the first unbiased proteome-wide view of a mammalian ADP-ribosylome, which includes the exact identification of endogenous ADP-ribose acceptor sites under different physiological conditions in cells and for the first time also in organ tissue. We show that PARG treatment of peptides before enrichment with Af1521, allows specific identification of ADP-ribosylated amino acids and highly sensitive identification of the corresponding proteins. And we demonstrate that the detected modifications are not derived from non-enzymatic glycation and, contrary to previous claims^50^, Af1521 does not hydrolyse the modification under the applied conditions. Besides, this method supports the identification of ADP-ribosylated proteins without complementing cell lysates or organelles with NAD^+^ analogues and mutated ARTDs (ref. 18) and is, consequently, able to analyse the ADP-ribosylome under different cellular conditions derived from endogenous ARTD protein and NAD^+^ levels in both cells and tissue.

The streamlined methodology led to the identification of more than 900 endogenous ADP-ribosylation sites belonging to more than 500 proteins in both culture and tissue cells. Our results suggest that this methodology could be used in combination with high-throughput screening techniques to identify endogenous proteins affected by ADP-ribosylation and/or those modulated by ADP-ribosylation inhibitors (also called PARP inhibitors). ARTD1 is a key regulator within the DDR (ref. 33), which has recently become a highly attractive target for cancer therapy^51^,^52^. Although not specific for ARTD1, several ADP-ribosylation inhibitors have been approved or are currently being evaluated in clinical trials as mono-therapeutic or combination therapy agents. Our ability to now analyse the endogenous ADP-ribosylome from different cancer cells or tissues constitutes a promising approach that should advance of our understanding of ADP-ribosylation in a clinical setting.

FEN1 is a structure-specific nuclease with 5′-flap endonuclease and 5′–3′ exonuclease activities involved in DNA replication and repair, and ARTD1 recruits FEN1 to DNA damage intermediates^53^. The K354 reside of FEN1 becomes acetylated through the acetyltransferase p300, which reduces the DNA-binding activity of FEN1 (ref. 54), while ubiquitylation of the same lysine mediates the proteosomal degradation of FEN1 during G2/M phase^55^. These PTM-based regulatory mechanisms support the notion that ADP-ribosylation at K354 might relate to a currently uncharacterized regulatory function of FEN1. The observed ADP-ribosylation of the SUMO-protein ligase CBX4 suggests nuclear cross-talk between PARylation and SUMOylation, which might be more widely occurring than previously anticipated. Such cross-talk could be analogous to previously reported ADP-ribosylation-dependent ubiquitylation (PARdu)^56^. In fact, CBX4 is known to mediate SUMO conjugation in the DDR, and the recruitment of CBX4 to sites of DNA lesions is dependent on ADP-ribosylation^57^. Thus, our observation that CBX4 becomes ADP-ribosylated during oxidative stress strongly suggests that we have identified a previously uncharacterized DDR regulatory mechanism controlled by PARylation-dependent SUMOylation (PARsu).

The presented methodology is suitable for the identification of key endogenous ADP-ribosylation events in biological processes. We find that ADP-ribosylated proteins are on average less modified under physiological conditions than reported under siPARG treatment^9^, which suggests that deregulation of PARG alters physiological ADP-ribosylation homoeostasis (Fig. 1b)^13^,^39^. Moreover, our MS analysis supports that Lys residues K498 and K505 are major acceptor sites in ARTD1, which follows previous mutational analysis of ADP-ribosylation sites on ARTD1 (ref. 7). Our analysis indicates that under physiological conditions ARTD1 is activated *in cis*^58^, contradicting the observed *in trans* activation of ARTD1 under siPARG treatment^9^.

Comparison of the SILAC and IF data obtained from HeLa cells revealed that the dynamic changes induced by H_2_O_2_ for these two data sets correlate well with each other. The relative increase in peptide abundance may thus suggest that the ADP-ribosylation sites detected reflect PARylation, at least partially. However, since the methodology described here cannot discriminate between MAR and PAR, each regulated ADP-ribosylation site will require follow-up experiments to determine whether they are indeed PARylated or MARylated.

The importance of tissue-specific protein ADP-ribosylation mapping is underscored by the substantial differences in ADP-ribosylation observed between cell culture and tissue (that is, liver). In mouse liver tissue the majority of ADP-ribosylated proteins localize to compartments containing enzymes with MAR activity, such as ARTD10, ARTD8, ARTC and SIRT4, suggesting that these proteins are primarily MARylated rather than PARylated (Fig. 4e). These observations are further supported by the increased levels of modified Arg residues, and membrane and extracellular proteins, which might stem from ARTC activity. Moreover, our data substantiates initial studies where ADP-ribosylation was observed on Arg residues within rat liver proteins^59^. From the analysed liver extracts, ARTD1 was not found to be auto-ADP-ribosylated, highlighting the fact that the enzyme is mainly inactive under normal, non-stressed conditions.

Our organ analysis of ADP-ribosylation provides evidence that the modification is involved in multiple physiological functions. For example, KEGG pathway analysis reveals that proteins involved in actin cytoskeleton and endoplasmic reticulum regulation are enriched in ADP-ribosylation (Supplementary Fig. 5c). These pathways have previously been associated with ADP-ribosylation^60^,^61^, but the mechanism by which the modifications are catalysed (that is, ARTDs or ARTCs (ref. 62)) remains to be investigated. NAD^+^ can be released on necrosis or mechanic stress in tissue, which in turn likely activates ARTCs during organ collecting^63^. Considering that ARTCs have higher affinity for NAD^+^ compared with ARTD1, this renders ARTCs more prone to activation following NAD^+^ release during tissue collecting. Moreover, we find that proteins involved in metabolic processes, oxidative-reduction processes and mitochondrial content are distinctly enriched in ADP-ribosylation targets (Fig. 4e and Supplementary Fig. 5f), which suggests these processes may be influenced by the modification. While the exact mechanisms are not well understood, inhibition of ARTDs is known to enhance these processes^64^. With ARTD1 activation known to affect cellular metabolism *via* direct PARylation events, transcriptional reprogramming, or alterations in cellular NAD^+^ levels^64^, our tissue analysis supports the hypothesis that metabolic consequences on ADP-ribosylation inhibition might occur via alterations in NAD^+^ levels^65^. This is based on ARTD1 being an avid NAD^+^ consumer, and that ADP-ribosylation inhibition increases the cellular levels of NAD^+^ available for other ARTDs (ref. 65), in a manner reminiscent of the reported interplay between ARTD1 activity and deacetylase SIRT1 (ref. 66). Furthermore, with immortalized cell lines exhibiting mitochondria and metabolic processes deficiencies^67^, hereby rendering them impractical for ADP-ribosylation inhibition/activation and cellular metabolism investigations, highlights that methodologies allowing proteomics-based whole tissue analyses are required to investigate this antagonistic interplay in more detail.

In conclusion, our novel methodology allows for the unbiased and sensitive characterization of ADP-ribosylation sites under physiological conditions, while the data presented here extends current ADP-ribosylation knowledge and highlights the widespread occurrence of the modification. Although the methodology presented cannot currently distinguish between MARylated and PARylated peptide species, adjustment of the binding reaction stringency and combining the enrichment with specific MAR-binding domains, will most likely facilitate dissecting peptide MAR- versus PARylation in a more specific manner^68^. Importantly, the approach presented supports comprehensive and quantitative evaluation of the mammalian ADP-ribosylome of cell lines and tissue samples. Thus, allowing downstream interrogation of disease pathways in which ARTs are implicated.

## Methods

### Cell culture and transfection

HeLa cells were grown in Dulbecco's modified Eagle's medium (D-MEM; Invitrogen) supplemented with 10% foetal bovine serum and penicillin/streptomycin (100 U ml^−1^) (Gibco). Stable HeLa-Kyoto cells expressing CBX4, SRSF2, SRSF1 and FUS tagged with C-terminal GFP under the control of an endogenous promoter were generated by transfecting BAC transgenes and were kindly provided by Prof Anthony Hyman (Max Planck Institute, Dresden). Selection was maintained by adding 400 μg ml^−1^ G418 (Sigma Aldrich) to the culture medium. SILAC HeLa cells were grown in SILAC D-MEM (Invitrogen) supplemented with 10% dialyzed foetal bovine serum, L-glutamine, penicillin/streptomycin, and either L-lysine and L-arginine, L-lysine 4,4,5,5-D4 and L-arginine-U-13C6, or L-lysine-U-13C6-15N2 and L-arginine-U-13C6-15N4 (Cambridge Isotope Laboratories)^36^. The siRNA oligonucleotides against endogenous PARG (ID: 4390826) was purchased from Ambion as well as Negative Control siRNA#1. SiRNA transfections were performed using Lipofectamine RNAiMAX (Invitrogen) according to the manufacturer's protocol and lysed 48 h after transfection. All HeLa cells used for experiments were tested negative for mycoplasma.

### Sample preparation

Cells were stimulated with H_2_O_2_ (Sigma Aldrich) for 10 min in PBS at 37 °C, collected by washing with ice-cold PBS and lysed in modified RIPA buffer (50 mM Tris pH 7.5, 400 mM NaCl, 1 mM EDTA, 1% Nonidet P-40, 0.1% Na-deoxycholate), protease inhibitor mixture (Roche) supplemented with 2 mM Na-orthovanadate, 5 mM NaF, 5 mM Glycero-2-phosphate, 1 μM ADP-HPD (Millipore) and 40 μM PJ-34 (Enzo Life Sciences) and cleared by high-speed centrifugation. Proteins were precipitated by adding fourfold excess volumes of ice-cold acetone and stored at −20 °C overnight. Subsequently, proteins were solubilized in a urea solution (6 M urea/2 M thiourea/10 mM HEPES pH 8.0). Protein concentrations in lysates were measured using Bradford assay (Bio-Rad). Next, proteins were reduced by adding dithiothreitol to a final concentration of 1 mM, and alkylated with chloroacetamide at 5.5 mM. Proteins were digested using endoproteinase Lys-C (1:100 w/w) and modified sequencing grade trypsin (1:100 w/w) after a fourfold dilution in 50 mM ammonium bicarbonate solution. Protease digestion was terminated by slow addition of trifluoroacetic acid to pH 2. Precipitates were removed by centrifugation for 10 min at 3,000*g*. Peptides were purified using reversed-phase Sep-Pak C18 cartridges (Waters). Peptides were eluted off the Sep-Pak with 50 and 80% acetonitrile.

### GST-protein expression and purification of Af1521

BL21 was used for long transformation. Briefly, 1 μl of cooled plasmid was added to BL21 and left on ice for 15 min. Bacteria were heat shocked at 42 °C for 45 sec, incubated for 1 min on ice and then mixed with SOC for 40 min at 37 °C. Bacteria were streaked onto Amp-plates and left overnight at 37 °C. The following day a single colony was inoculated in LB media and grown overnight at 37 °C. The starter culture was diluted and grown to an OD600 of 0.55–0.65. Protein expression was induced by adding IPTG to final concentration of 0.5 mM and incubated for 5–6 h. Bacteria were spun down and pellet frozen at −80 °C.

The bacterial pellet was thawn and incubated for 20 min in lysis buffer (50 mM Tris–HCl, pH 7.5, 150 mM NaCl, 1 mM MgCl2, 1 mM dithiothreitol, 1 × Bug Buster (Novagen), 1 μl ml^−1^ Benzonase (Sigma Aldrich), 200 μg ml^−1^ lysozyme (Sigma Aldrich), protease inhibitor mixture (Roche)). After breaking cells by vortexing with glass beads, cell debris was pelleted by centrifugation. The cleared lysate was incubated for 4 h at 4 °C rolling with equilibrated glutathione sepharose 4B (Sigma Aldrich). Beads were washed four times in wash buffer (50 mM Tris–HCl, pH 7.5, 150 mM NaCl and 1 mM dithiothreitol), resuspended in wash buffer and kept at 4 °C for up to 3 weeks.

### Enrichment of ADP-ribosylated peptides

After eluting off Sep-Pak, appropriate amounts of IP buffer (50 mM Tris–HCl, pH 8, 10 mM MgCl2, 250 μM dithiothreitol and 50 mM NaCl) was added before the acetonitrile and the volume was reduced by vacuum centrifugation. PAR complexity was reduced by incubation with PARG (4.2 μg per sample) for 3 h at 37 °C. The peptide mixture was cooled down before it was incubated rotating for 2 h at 4 °C with the purified Af1521 macro domain. The peptides were washed three times in ice-cold IP buffer followed by one wash in water, and modified peptides were eluted with 2 × 100 μl 0.15% TFA in Milli-Q water. Peptide eluates were desalted on reverse phase C18 StageTips^69^.

### Mass spectrometric analysis

All MS experiments were performed on a nanoscale EASY-nLC 1000 UHPLC system (Thermo Fisher Scientific) connected to an Orbitrap Q-Exactive Exactive equipped with a nanoelectrospray source (Thermo Fisher Scientific). Each sample was eluted off the StageTip, auto-sampled and separated on a 15 cm analytical column (75 μm inner diameter) in-house packed with 1.9-μm C18 beads (Reprosil Pur-AQ, Dr Maisch) using a 3 h gradient ranging from 5 to 64% acetonitrile in 0.5% formic acid at a flow rate of 200 nl min^−1^. The effluent from the high-performance liquid chromatography was directly electrosprayed into the mass spectrometer. The Q Exactive Plus mass spectrometer was operated using data-dependent acquisition, with all samples being analysed using a ‘sensitive' acquisition method^22^ and a normalized collision energy of 28. Back-bone fragmentation of eluting peptide species were obtained using HCD which ensured high-mass accuracy on both precursor and fragment ions.

### Mass spectrometry analysis of ADP-ribosylation sites by ETD

ETD spectra of ADP-ribosylated peptides were acquired on an Orbitrap Fusion Lumos mass spectrometer (Thermo Scientific) operating in positive ion mode. Full MS scans (*m/z* 300–1,500) were performed at 120,000 resolution (*m/z* 200) in the Orbitrap, with the AGC target set at 4e5. Precursor selection was prioritized on the basis of highest charge state followed by highest intensity. Peptides (charge states from 3+ to 6+) were selected by the quadrupole (1.3 *m/z* isolation window) before reaction with fluoranthene radical anions (ETD reagent target 4e5). ETD reaction times were set at 1.7τ for each charge state. MS/MS spectra were acquired using a normal ion trap scan rate with a maximum injection time of 50 ms (AGC target 2e5). A Venn diagram comparing identified protein targets derived from the ETD with HCD analysis is shown in Supplementary Fig. 6a.

### *In vitro* TOF-MS analysis of H2B peptide sequence

1 μg of HK326 peptide (PQPAKSAPAPKKG) was incubated with 1 mM ADP-ribose in 50 mM sodium phosphate buffer (pH 7.5 and pH 9.5) for 1 h or overnight at 37 °C. Samples were desalted using Reversed-phase m-C18 ZipTips (for MALDI-MS) and eluted with MALDI matrix solution (a-cyano-4-hydroxycinnamicacid in 0.3 mM di-ammonium hydrogen citrate (Fluka), 60% acetonitrile in H_2_O) directly on the target plate. MALDI analyses were performed on a 4800 MALDI TOF/TOF system in linear mode.

### Identification of peptides and proteins

All raw data analysis was performed with MaxQuant software suite version 1.3.0.5 supported by the Andromeda search engine^70^. Data were searched against a concatenated target/decoy (forward and reversed) version of the UniProt Human fasta database encompassing 71,434 protein entries (downloaded from www.uniprot.org on 2013-07-03). Mass tolerance for searches was set to maximum 7 p.p.m. for peptide masses and 20 p.p.m. for HCD fragment ion masses. Data were searched with carbamidomethylation as a fixed modification and protein *N*-terminal acetylation, methionine oxidation and mono-ADP-ribosylation (*m/z* 541,06110: C10H13N5O9P2) on lysine, arginine, glutamic and aspartic acids as variable modifications. A maximum of three mis-cleavages was allowed while requiring strict trypsin specificity, and only peptides with a minimum sequence length of seven were considered for further data analysis. Peptide assignments were statistically evaluated in a Bayesian model on the basis of sequence length and Andromeda score. Only peptides and proteins with a false discovery rate of <1% were accepted, estimated on the basis of the number of accepted reverse hits, and false discovery rate values were finally estimated separately for modified and unmodified peptides. Protein sequences of common contaminants such as human keratins and proteases used were added to the database. For SILAC quantification a minimum of two ratio-counts was required.

### Enrichment of GFP-tagged and ADP-ribosylated proteins

Cells expressing the tagged versions of the proteins of interest were collected by washing with PBS and lysed in modified RIPA buffer (50 mM Tris pH 7.5, 400 mM NaCl, 1 mM EDTA, 1% Nonidet P-40, 0.1% Na-deoxycholate), protease inhibitor mixture (Roche) supplemented with 2 mM Na-orthovanadate, 5 mM NaF, 5 mM Glycero-2-phosphate, 1 μM ADP-HPD (Millipore) and 40 μM PJ-34 (Enzo Life Sciences). Lysates were diluted in modified RIPA without salt and then cleared by high-speed centrifugation.

GFP-immunoprecipitaion was performed with 20 μl GFP-Trap_A agarose beads (Chromotek). 1 mg of protein mixtures were incubated for 2 h rotating at 4 °C before washing and subsequent elution with 2 × Laemmli sample buffer (Thermo Fisher Scientific) at 90 °C. Pull down of ADP-ribosylated proteins was performed similarly but using 200 μl crosslinked Af1521 macro domain and 2 mg of protein mixtures.

### Western blotting

The following antibodies were used in this study: rabbit polyclonal PAR 1:1,000 (ALX-210–890A, Enzo Life Science) and mouse monoclonal GFP 1:1,000 (11814460001, Roche).

Total cell lysates together with the eluates were resolved on 4–12% gradient SDS–PAGE gels (Thermo Fisher Scientific) and proteins were transferred onto nitrocellulose membranes (Sigma Aldrich). Membranes were blocked using 5% BSA solution in PBS supplemented with Tween-20 (0.1%). Secondary antibodies coupled to horseradish peroxidase (Jackson ImmunoResearch Laboratories) were used for immunodetection. The detection was performed with Novex ECL Chemiluminescent Substrate Reagent Kit (Invitrogen). For slot blot analysis, PAR polymer (Trevigen) was incubated with different concentrations of PARG and spottet directly onto PVDF membranes (Millipore) using the slot blot chamber (Fisher Scientific) according to manufacturer's protocol. Cropped WBs presented in Fig. 2d have been included as uncropped scans in Supplementary Fig. 6b.

### Immunofluorescence microscopy

HeLa cells were seeded on coverslips and the following day stimulated for indicated time points with H_2_O_2_. After washing with PBS, cells were fixed in methanol/acetic acid solution and incubated for 5 min at 37 °C. Coverslips were blocked in 5% milk powder, transferred to a humid chamber and incubated with rabbit polyclonal PAR (Enzo Life Science) for 1 h at 37 °C and with secondary antibody Alexa Fluor 488 (Invitrogen) for 1 h at 37 °C, stained with DAPI for 2 min, washed in PBS and mounted. Images were acquired on a DFC345 FX microscope (Leica) and analysed using ImageJ.

### Immuno-slot-blot

For the immuno-slot-blot analysis, HeLa cells were treated and lysed as described in sample preparation, and proteins were vacuum aspirated onto a Hybond P 0.2 PVDF (Amersham Biosciences) using a slot-blot manifold (Amersham Biosciences). The membrane was blocked with 5% milk powder in 10 mM Tris–HCL (pH 8.0), 150 mM NaCl and 0.05% (v/v) Tween 20 (TBST buffer) and incubated with polyclonal PAR antibody (Enzo Life Science) diluted 1:1,000 in 5% milk powder in TBST for 1 h at RT and with secondary antibody IRDye 800CW goat anti-rabbit IgG (LI-COR) 1:15,000 in TBST for 1 h at RT. Signals were detected by the Odyssey infrared imaging system (LI-COR) and the immunoblot signal was quantified using GelEval (FrogDance Software).

### *In vitro* radiography assay

10 pmol of ARTD1 and ARTD10 were automodified with 100 nM NAD^+^ for 10 min at 37 °C, respectively, hereby inducing short PAR chains on ARTD1 and MAR on ARTD10 through auto-catalysis^29^. Samples were filtered through G50 columns to remove excess amount of unincorporated NAD^+^, and subsequently treated with 10 pmol PARG for additional 1 h at 37 °C. Using autoradiography assays the hydrolysis of attached radioactive 32 P-NAD^+^ of the protein substrates were subsequently monitored. For transmodification reactions, 50 pmol of target protein was incubated with 10 pmol ARTD1 for 10 min at 37 °C. The reaction was stopped by adding Laemmli buffer and boiling for 5 min at 95 °C. After separation of samples by SDS–PAGE, the radiolabelled ADP-ribosylation signal was determined by GelEval (FrogDance Software).

### Preparation of mouse liver extracts

Male 9-week-old C57BL/6 mice were maintained on a 12-h light–dark cycle with regular unrestricted diet. Mice were killed in a CO_2_ chamber. Excised livers were washed in PBS and shock frozen in liquid nitrogen. Ice-cold modified RIPA buffer (supplemented with PARP-, PARG- and protease inhibitors as described before) was added to the shock frozen livers and they were lysed operating the Tissue Lyser II (Qiagen) device at 30 HZ for 4 × 30 s. The lysate was further sonicated until it became fluid and all liver pieces were dissolved. The lysate was cleared by high-speed centrifugation and processed similar as described for the cell culture samples. 20 mg of liver protein was used as starting material for the digest.

### Bioinformatic analyses

Statistical analysis and hierarchical clustering was performed using the Perseus software suite (Max Planck Institute of Biochemistry, Department of Proteomics and Signal Transduction, Munich). Significantly enriched Gene Ontology terms were determined using the Functional Annotation Tool of the DAVID Bioinformatics database. Protein interaction networks were analysed using the interaction data from the STRING database (v. 9.05) and visualized using Cytoscape (v. 2.8.3). Protein abundance assessment was performed using a deep proteome reference data set for HeLa cells. All Venn diagrams were generated using the online Venny program (http://bioinfogp.cnb.csic.es/tools/venny/).

### Comments related to animal study

C57BL/6J mice were bread at the animal facility of the University of Zurich. No randomization or blinding were used for these studies, and no animals had to be excluded. All animal experiments were carried out in accordance with the Swiss and EU ethical guidelines and have been approved by the local animal experimentation committee of the Canton of Zurich under licence #2012207 and following the 3R guidelines.

### Data availability

The mass spectrometry proteomics data have been deposited to the ProteomeXchange Consortium via the PRIDE partner repository^71^ with the data set identifier PXD004245. The additional data that support the findings of this study are available from the corresponding author on request.

## Additional information

**How to cite this article:** Martello, R. *et al*. Proteome-wide identification of the endogenous ADP-ribosylome of mammalian cells and tissue. *Nat. Commun.* **7,** 12917 doi: 10.1038/ncomms12917 (2016).

## Supplementary Material

Supplementary Information Supplementary Figures 1 - 6 and Supplementary Note 1

Supplementary Data 1 Identified ADP-ribosylation sites in triplicate HeLa analyses. List of all identified ADP-ribosylation sites from three replicate analyses in HeLa cells. The first Excel sheet provides all identified sites with a localization probability of more than 0.60. Table is provided as an Excel file in the online additional supplementary materials.

Supplementary Data 2 Identified ADP-ribosylation sites in HeLa cells with ETD fragmentation. List of all identified ADP-ribosylation sites with a localization probability of more than 0.60 identified using ETD fragmentation in HeLa cells. Table is provided as an Excel file in the online additional supplementary materials.

Supplementary Data 3 Identified ADP-ribosylation sites - In vitro PAR analysis - Forward+Reverse. List of all identified ADP-ribosylation sites with a localization probability of more than 0.60. Forward and reverse SILAC ratios are marked in green. Table is provided as an Excel file in the online additional supplementary materials.

Supplementary Data 4 ADP-ribosylation occupancy values. List of calculated stoichiometry values in HeLa cells. Stoichiometry values describe the fraction of a protein that is modified at a given modification site and are calculated based on the ADP-ribosylated peptide ratio (marked in green), the unmodified peptide ratio (marked in blue) and the protein ratio (marked in orange). Stoichiometry values are listed for both light and heavy SILAC conditions. Table is provided as an Excel file in the online additional supplementary materials.

Supplementary Data 5 Identified ADP-ribosylation sites in mouse liver tissue. List of all identified ADP-ribosylation sites with a localization probability of more than 0.60 from three replicate analyses in mouse liver tissue. Table is provided as an Excel file in the online additional supplementary materials.
