# Supplementary material for: Proteome-wide identification of the endogenous ADP-ribosylome of mammalian cells and tissue
Source: Nat Commun. 2016 Sep 30;7:12917. doi: 10.1038/ncomms12917 (PMC5056437; doi:10.1038/ncomms12917)
Supplement: Supplementary Information — Supplementary Figures 1 - 6 and Supplementary Note 1 [file ncomms12917-s1.pdf]

Supplementary Figure 1

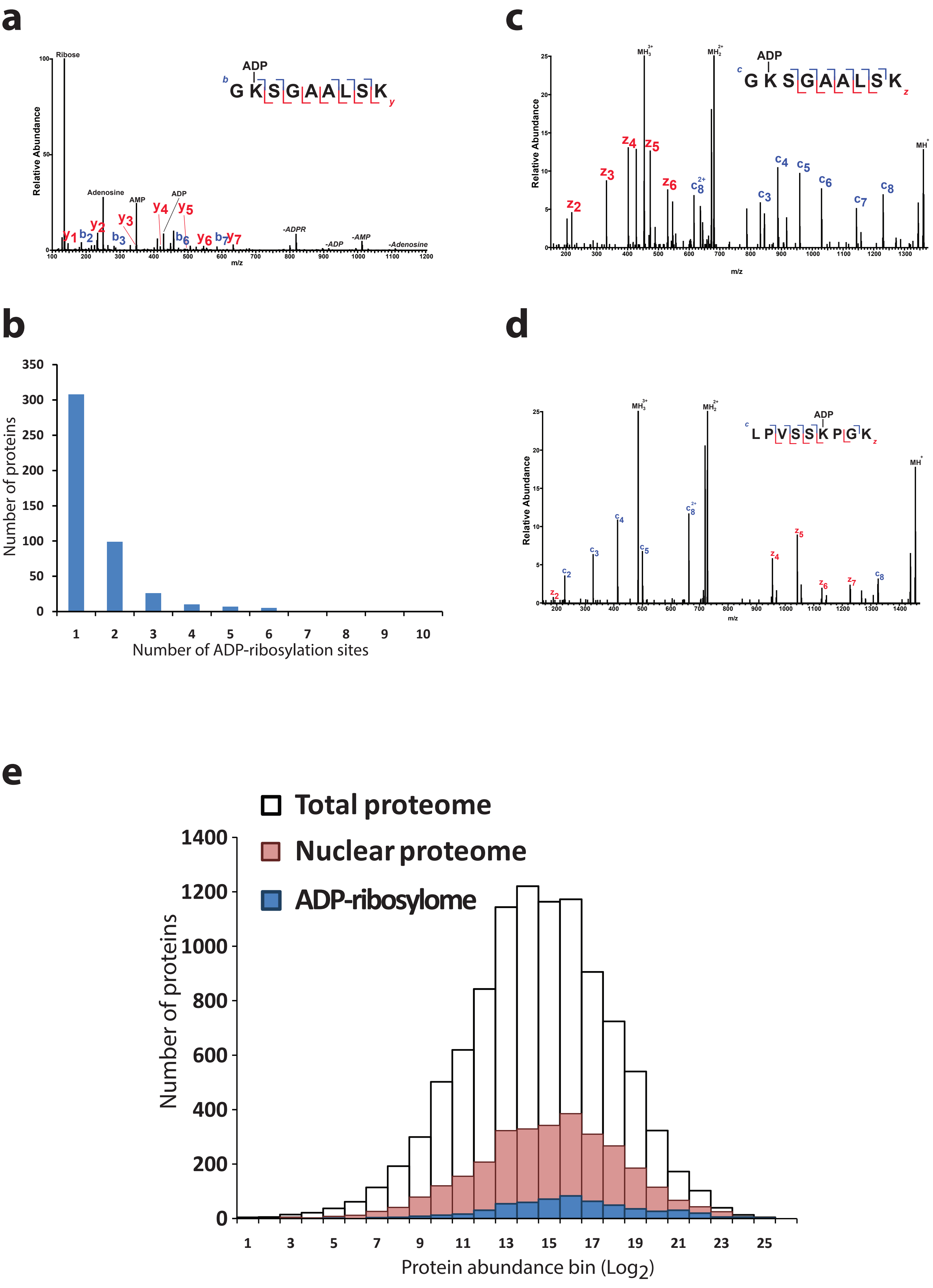

Supplementary Figure 1

(a) HCD tandem mass spectrum of peptide sequence GKSGAALSK, identifying Lysine K498 as ADP-ribosylated acceptor site. Identification of ADP-ribosylated peptides are greatly aided by the diagnostic ions generated by the ADP-ribosylation group of the modified peptide (Labeled as “Ribose”, “Adenosine”, “AMP”, “ADP”, “-ADPR”, “-ADP”, “-AMP”, “-Adenosine”.

(b) Distribution of identified ADP-ribosylation sites per protein

(c) ETD tandem mass spectrum of peptide sequence GKSGAALSK, idenfying Lysine K498 as ADP-ribosylated acceptor site. Further corroborating the ability of HCD for mapping of ADP-ribosylation sites.

(d) ETD tandem mass spectrum of peptide sequence LPVSSKPGK.

(e) Abundance distribution of the human HeLa proteome (white bars); the distribution of the annotated human nuclear HeLa proteome (red bars); and the distribution of identified ADP-ribosylated factors (blue bars).

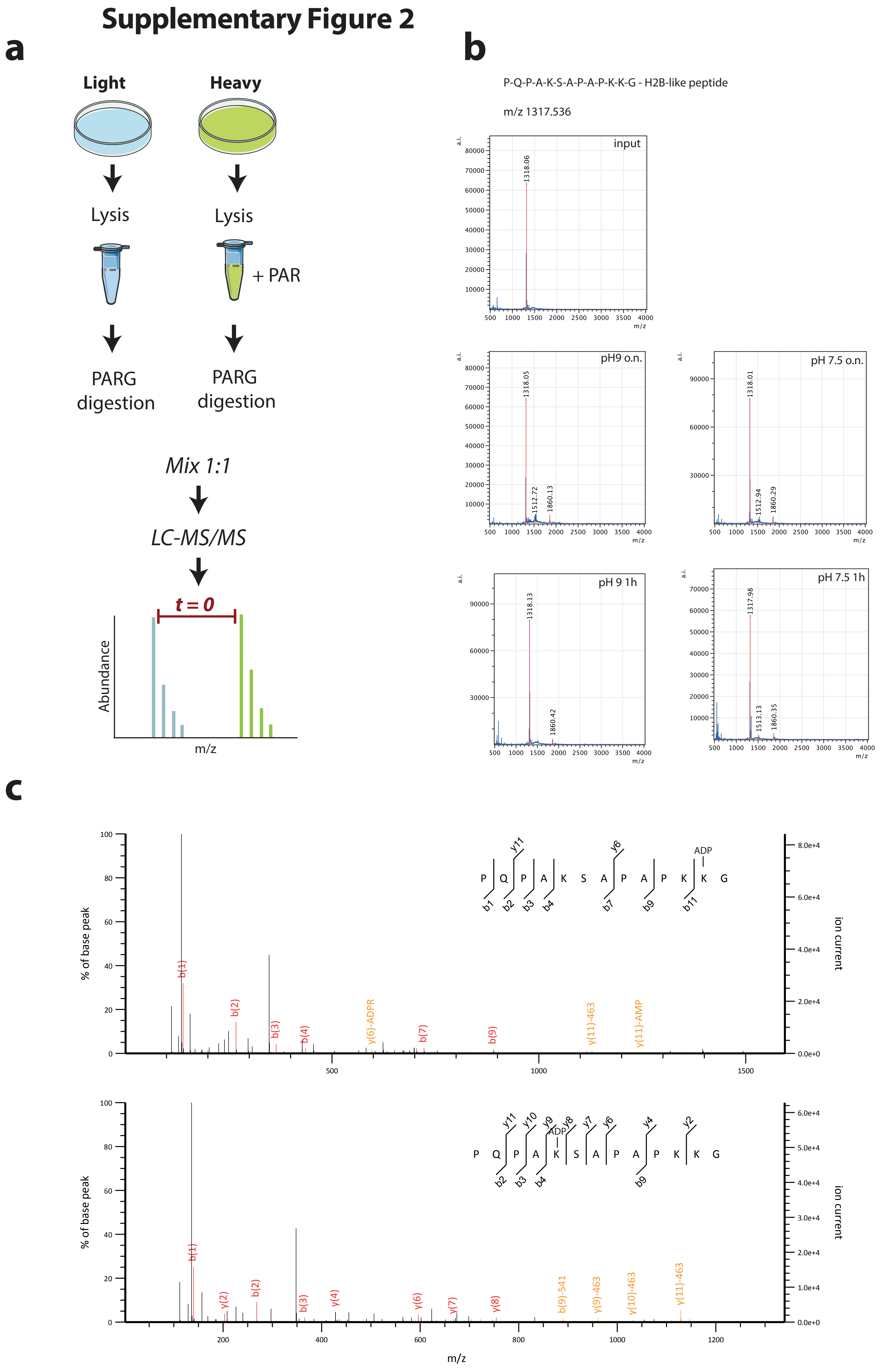
Supplementary Figure 2
a
Light
Heavy
Lysis
Lysis
+ PAR
PARG digestion
PARG digestion
Mix 1:1
LC-MS/MS
t = 0
Abundance
m/z
b
P-Q-P-A-K-S-A-P-A-P-K-K-G - H2B-like peptide
m/z 1317.536
input
1318.06
pH9 o.n.
1318.05
1512.72
1860.13
pH 7.5 o.n.
1318.01
1512.94
1860.29
pH 9 1h
1318.13
1860.42
pH 7.5 1h
1317.98
1513.13
1860.35
c
% of base peak
ion current
m/z
P Q P A K S A P A P K K G
ADP
b1 b2 b3 b4 b7 b9 b11
y11 y6
b(1) b(2) b(3) b(4) y(6)-ADPR b(7) b(9) y(11)-463 y(11)-AMP
y11 y10 y9 y8 y7 y6 y4 y2
b2 b3 b4 b9
b(1) y(2) b(2) b(3) y(4) y(6) y(7) y(8) b(9)-541 y(9)-463 y(10)-463 y(11)-463

Supplementary Figure 2

(a) Experimental design of SILAC experiment to determine if PARG digestion is able to induce in vitro glycation. Briefly, heavy labeled SILAC cells were treated with purified PAR while light SILAC cells were left untreated. Both cell conditions were subsequently treated with PARG, mixed 1:1 and ADP-ribosylated peptides analysed by LC-MS/MS. If PARG digestion is able to cause in vitro glycation we would expect identification of SILAC regulated ADP-ribosylation sites.

(b) In vitro glycation analysis of H2B peptide sequence PQPAKSAPAPKKG at different time-points and pH.

(c) Tandem mass spectrum that map in vitro glycations products to lysine residues within the investigated H2B peptide, PQPAKSAPAPKKG.

Supplementary Figure 3

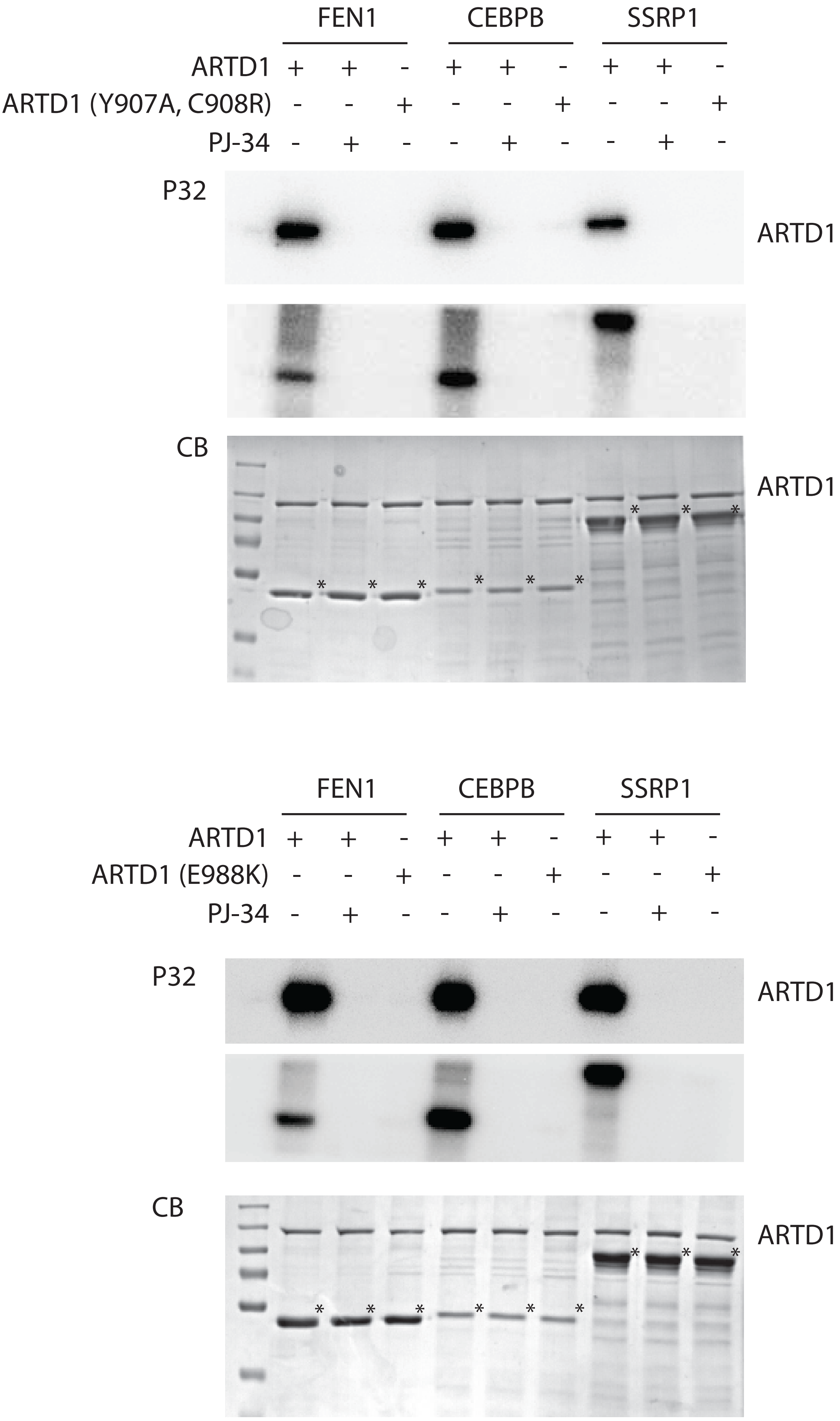

Supplementary Figure 3

In vitro PARylation analysis of identified protein targets FEN1, CEBPB and SSRP1. Various inactive mutant versions of purified full-length human ARTD1 (Y907A, C908R or E988K) was incubated with recombinantly expressed proteins in the presence of 32P-NAD and double-stranded DNA oligomer. Samples were resolved by SDS-PAGE, stained with Coomassie (CB; lower panels) and 32P-incorporation was detected by autoradiography (P32; upper panels).

For all investigated PARP1 mutants no ADP-ribosylation was detected, confirming that the ADP-ribosylation observed (Figure 2c) is catalyzed by wild-type PARP1 and not a glycation product.

Supplementary Figure 4

a

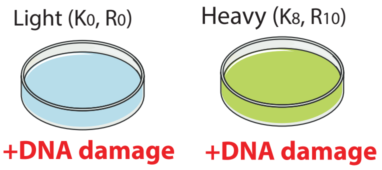

Experiment	Light SILAC	Heavy SILAC
1	0 Min	0 Min
2	0 Min	5 Min
3	0 Min	10 Min
4	0 Min	30 Min
5	0 Min	60 Min
6	0 Min	120 Min

b

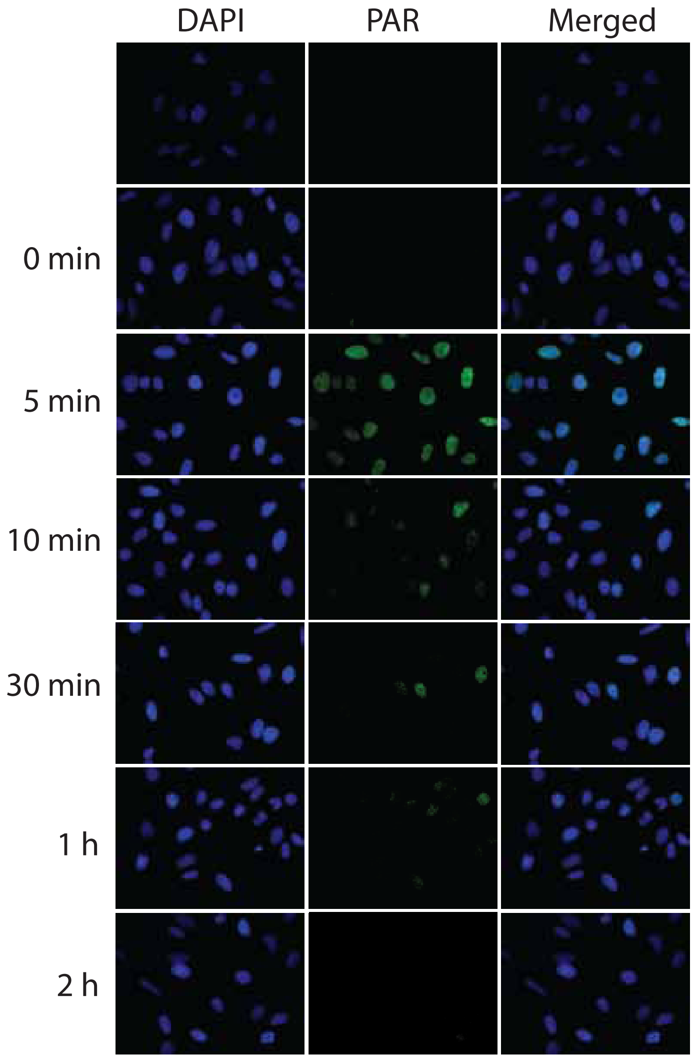

c

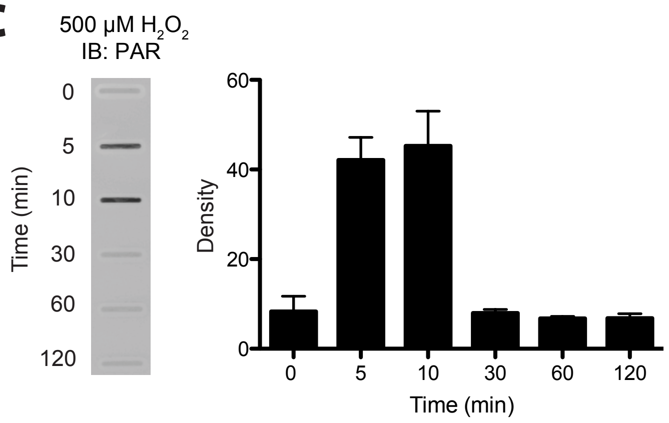

d

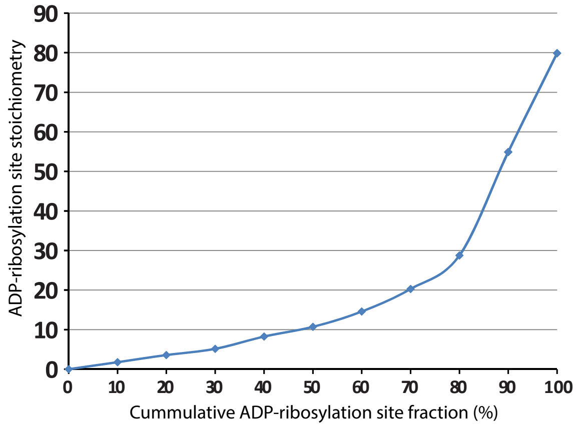

e

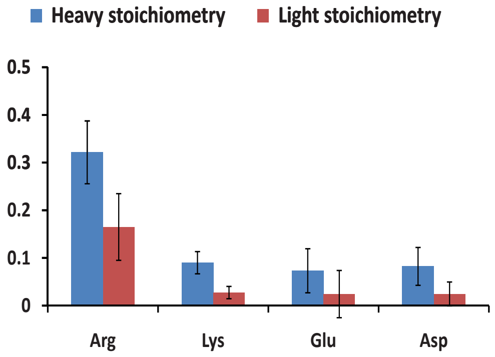

f

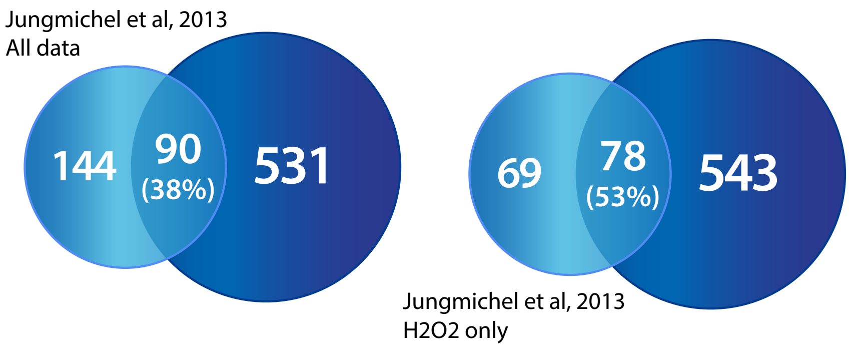

g

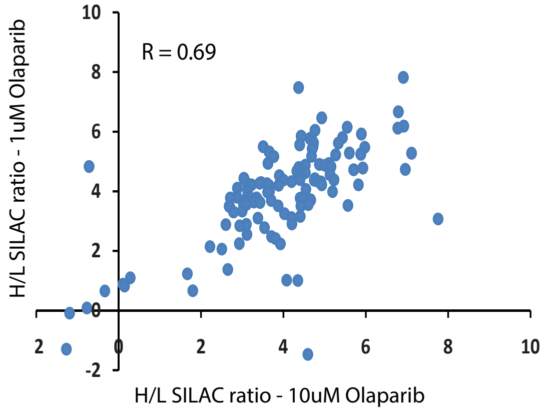

Supplementary Figure 4

(a) Experimental design used for quantitative evaluation of ADP-ribosylation sites (SILAC ratios). Light SILAC condition was exposed shortly to H_2O_2 while heavy SILAC was exposed as indicated. A quantitative analysis revealed that measured SILAC ratios are most increased following 5-10 stimulation with H_2O_2.

(b) Immunoflourescence (IF) imaging of cells treated with H_2O_2 for indicated time-points (0 min, 5 min, 10 min, 30 min, 1 hour, 2 hours), and immunostained with PAR-specific antibody or DAPI as indicated. Strongest PAR signal is observed after 5-10 minute treatment of H_2O_2.

(c) Left: Immuno-slot blot analysis of PAR formation in HeLa cells treated with H_2O_2 for indicated time-points (0 min, 5 min, 10 min, 30 min, 1 hour, 2 hours). Right: Strongest PAR signal is observed after 5-10 minute treatment of H_2O_2.

B) Densiometric evaluation of immune-slot blot analysis. The strongest abundance in PAR signal is observed after 5-10 minutes treatment of H_2O_2. Data represent mean +/- SD of n=3 independent analyses.

(d) Distribution of ADP-ribosylation occupancy in cells exposed to H_2O_2 for 10 minutes. Fifty percent of all ADP-ribosylation sites have occupancy of 11% or more.

(e) Distribution of amino acid occupancies across identified ADP-ribosylation sites in cells exposed to H_2O_2 for 10 minutes. Occupancy is determined for both heavy (Blue bars) and light (Red bars) SILAC conditions, which correspond to treated and untreated conditions, respectively. Collectively arginine residues are observed harboring the highest occupancy.

(f) Venn diagram between identified ADP-ribosylated substrates and previously identified PARylated substrates derived from Jungmichel et al, 2013.

(g) Scatter plot show correlation of logarithmized H/L ratios from Olaparib treated SILAC experiments as indicated.

Supplementary Figure 5

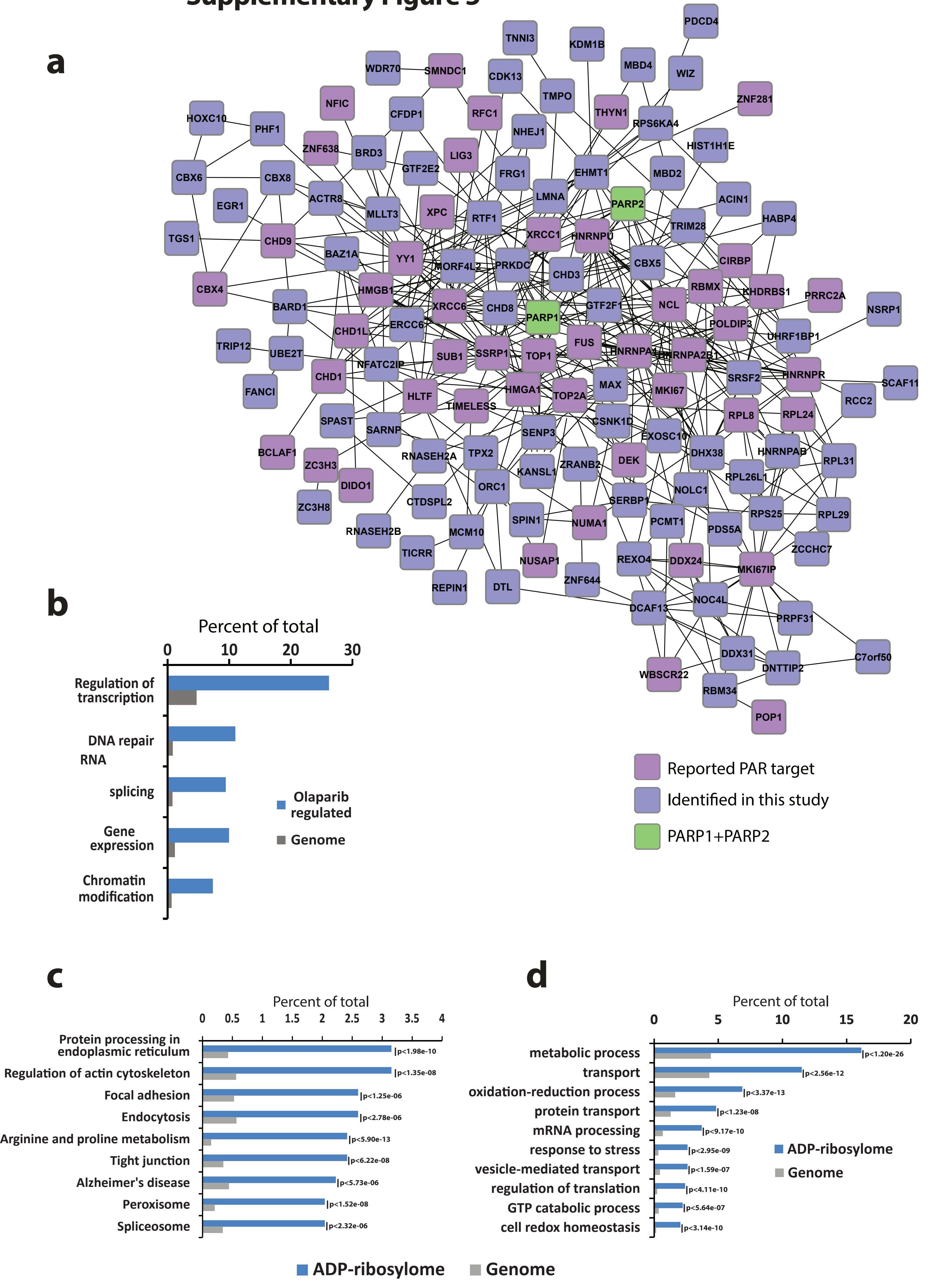

Supplementary Figure 5

(a) Protein interaction networks of regulated ADP-ribosylation sites upon Olaparib treatment. Data was combined from two SILAC experiments where cells were treated with two different inhibitor concentrations (1 µM and 10 µM Olaparib). Network interaction data was extracted from the STRING database and visualized using Cytoscape.

(b) GO functional annotation of significantly regulated proteins from Olarapib SILAC experiments as compared to annotated GO genes in the entire genome (indicated p-values < 1.5e-17). Strong enrichment for biological processes known to be targeted by PARP is observed.

(c) GO biological processes annotation of tissue-derived ADP-ribosylation factors compared to annotated GO genes across the entire human genome.

(d) KEGG pathway annotation of tissue-derived ADP-ribosylation factors compared to annotated genes across the entire human genome.

Supplementary Figure 6

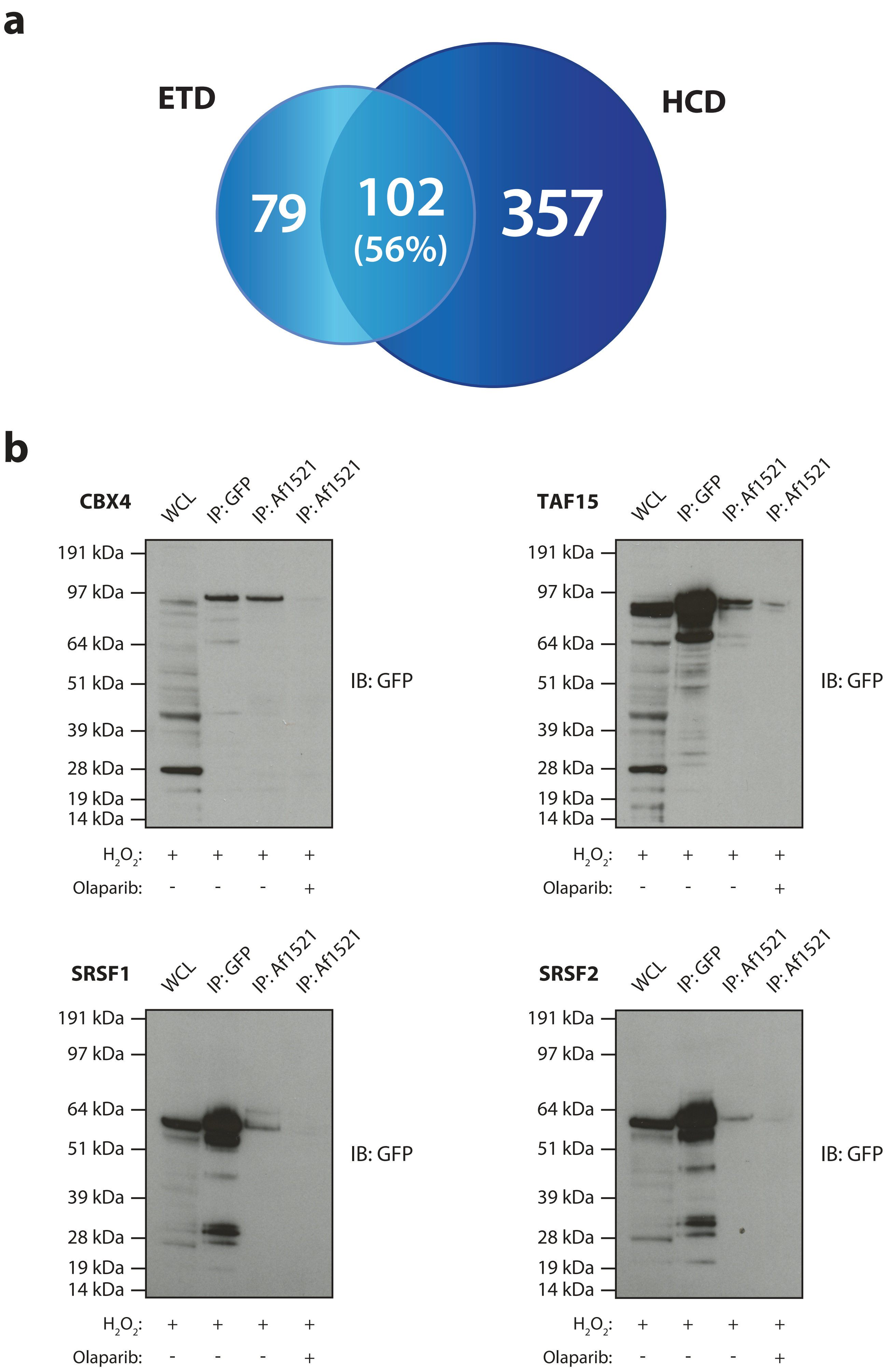

Supplementary Figure 6

(a) Venn diagram showing the overlap in identified proteins from analyzing Af1521 enrichment samples with HCD versus ETD (comparison of data listed in Supplementary Data 1 and Supplementary Data 5).

(b) Uncropped western blots from Fig. 2d.

Supplementary Note 1 - Estimation of enrichment level

In order to establish a proper lysates:Af1521 level, we incubated a fixed amount of purified Af1521 macrodomain with increasing amounts of a H2O2-treated HeLa lysates (2.5 mg, 5 mg, 10 mg, 15 mg, 20 mg of lysate material, respectively). Following incubation and Af1521 enrichment, we determined the number of identified ADP-ribosylation sites by LC-MS analysis (Figure a). From replicate analyses we observed an increasing amount of identified ADP-ribosylation sites with increasing levels of HeLa lysate. However, a saturation level was observed at levels above 10 mg of cell lysates (Figure a), indicative of saturation of the Af1521 domain. Hence, from these data we determined that the appropriate amount of cell lysate material for our enrichment procedure would be 8-10 mg.

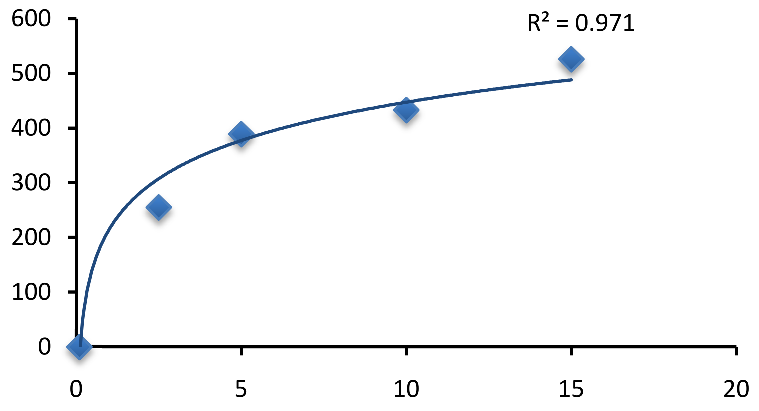

Figure a

To estimate the enrichment level of the developed methodology, we compared the number of identified ADP-ribosylation sites from a non-enriched HeLa cell lysate to the same sample after enrichment with our Af1521 methodology. Both samples were initially treated with 500 µM H_2O_2 to induce formation of ADP-ribosylation, and subsequently both samples were treated with PARG enzyme to convert all PAR into MAR.

However, in the next step, only one lysate sample was used for enrichment of ADP-ribosylation sites using the Af1521 macrodomain while the other whole HeLa cell lysate was left non-enriched. Both samples were subsequently analyzed by LC-MS using identical LC gradient and MS settings, and the obtained data files were processed for identification of ADP-ribosylation sites using the MaxQuant software suite (see method section for further details and settings).

From the LC-MS analysis of the non-enriched whole HeLa lysate (a total of 500 ng lysate was loaded onto the LC column) we identified only a single (1) ADP-ribosylation site, which is in stark contrast to the >500 ADP-ribosylation sites identified using the Af1521 enrichment methodology under identical LC-MS settings (similar LC gradient length and MS acquisition method). To compare this to another PTM, we additionally searched the non-enriched whole HeLa lysate for phosphorylations from which we identified 70 high-confident phosphorylation sites (all with localization score >0.75).

From these data we conclude our Af1521 methodology entails an enrichment level of minimum 500.

	Total peptides	ADP-ribosylated peptides	Phosphorylated peptides
**500 ug whole HeLa cell lysate**	~25,000	1	70
**Af1521 enrichment – 2.5mg**	15,814	244	-
**Af1521 enrichment – 5mg**	19,122	395	-

**Af1521 enrichment – 10mg**	19,039	433	-
**Af1521 enrichment – 15mg**	18,484	526	-
